# The DIRAC framework: Geometric structure underlies roles of *diversity* and *accuracy* in combining classifiers

**DOI:** 10.1016/j.patter.2024.100924

**Published:** 2024-02-05

**Authors:** Matthew J. Sniatynski, John A. Shepherd, Lynne R. Wilkens, D. Frank Hsu, Bruce S. Kristal

**Affiliations:** 1 Division of Sleep and Circadian Disorders, Department of Medicine, Brigham and Women’s Hospital, Boston, MA 02115, USA; 2 Division of Sleep Medicine, Department of Medicine, Harvard Medical School, Boston, MA 02115, USA; 3 School of Medicine, University of California, San Francisco, San Francisco, CA, USA; 4 University of Hawaii Cancer Center, University of Hawaii at Mānoa, Honolulu, HI, USA; 5 Department of Computer and Information Science, Fordham University, New York, NY 10023, USA

**Keywords:** DIRAC, information fusion, system fusion, ranks, permutahedron, diversity, accuracy, correlation

## Abstract

Combining classification systems potentially improves predictive accuracy, but outcomes have proven impossible to predict. Similar to improving binary classification with fusion, fusing ranking systems most commonly increases Pearson or Spearman correlations with a target when the input classifiers are “sufficiently good” (generalized as “***accuracy***”) and “sufficiently different” (generalized as “***diversity***”), but the individual and joint quantitative influence of these factors on the final outcome remains unknown. We resolve these issues. Building on our previous empirical work establishing the DIRAC (*DI**versity* of Ranks and *AC**curacy*) framework, which accurately predicts the outcome of fusing binary classifiers, we demonstrate that the DIRAC framework similarly explains the outcome of fusing ranking systems. Specifically, precise geometric representation of ***diversity*** and ***accuracy*** as angle-based distances within rank-based combinatorial structures (permutahedra) fully captures their synergistic roles in rank approximation, uncouples them from the specific metrics of a given problem, and represents them as generally as possible.

## Highlights

• DIRAC’s prediction of fusion outcomes extended from binary to ordinal classification
• Quantitative demonstration of the joint influence of ***accuracy*** and ***diversity*** on outcome
• System-level fusion is fully predictable given characterization of the input models
• Theoretical underpinnings for pairwise system fusion of ranks are fully elucidated

## The bigger picture

Directly combining data models (i.e., system-level fusion) for binary classification or ranking offers an orthogonal approach to complement and potentially enhance standard modeling at a qualitatively different level, but this approach has been historically unpredictable in the absence of additional validation data. This fundamental technological gap limits our ability to leverage data across any domain in which more than one potentially useful model exists. The authors’ prior work in binary classification empirically demonstrated a relationship between input model accuracy, inter-model diversity, and fusion success, but key questions remained regarding the broader applicability of this relationship and the underlying theoretical foundation. A general solution that is domain and distribution independent will be broadly useful in critical real-world applications, such as biomarker development, personalized health, clinical trial enrollment, equity issues in data science, and portfolio assembly.

## Graphical abstract

**Figure:**
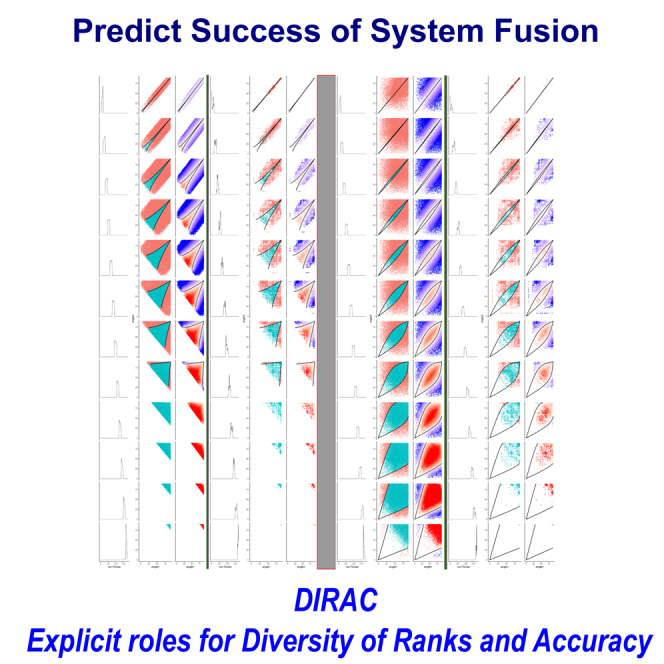

## Teaser

Combining ordinal classifiers potentially improves accuracy, but outcomes are not generally predictable. Fusing ordinal classifiers most commonly increases correlation with a target when the input classifiers are sufficiently “***accurate***” and “***diverse***,” but their individual and joint quantitative influence on fusion outcomes remains unknown. Here, the authors resolve these issues by formulating a precise geometric representation of their DIRAC framework, which they originally developed in empirical work to predict the outcome of fusing binary classifiers.

## Introduction

The integration of distinct and/or disparate data sources/types and the mathematical models that seek to explain and leverage these sources of information will be a primary creator of actionable knowledge across many fields. The general term “information fusion” collectively refers to these integration processes, of which there are many specific and familiar instances ranging, for example, from the averaging of multiple measurements to enhance reliability to the combination of many individual decisions into an optimal group decision (e.g., voting). The information sources to be integrated may provide similar information, such as the signals from redundant temperature sensors, or categorically different information, such as the MRI scan images and family disease histories of a health care system’s patients. The accuracy of these information sources may or may not be known, and the information that they provide may be identical, complementary, or contradictory.^1^^,^^2^^,^^3^^,^^4^^,^^5^^,^^6^^,^^7^^,^^8^^,^^9^

There are three generally recognized categories of information fusion that are distinguished on the basis of when they are applied during the transition from raw input data to final output decision.^10^^,^^11^^,^^12^^,^^13^ Operationally and for the sake of semantic clarity, we refer to these three categories as (1) data/feature fusion, (2) decision/prediction fusion, and (3) system fusion. “Data/feature fusion” refers to all fusion events that occur prior to the final fitting of mathematical models.^11^^,^^14^^,^^15^ “Data fusion” refers, for example, to the concatenation of multiple isomers and adducts in raw mass spectrometry data prior to data reduction. Data reduction then converts these multiple individual data items to a feature, e.g., a lipid. Feature fusion, which we include as an aspect of data fusion, then brings these variables (e.g., lipid 1, lipid 2) together to create a larger dataset for modeling or other uses. As such, data/feature fusion may be viewed conceptually as including all operations equivalent to the concatenation of two or more datasets. As such, data/feature fusion may encompass a broad range of specific fusion types, including, for example, the accrual of data from multiple, identical sensors that measure a single parameter (e.g., temperature) to the aforementioned example in which MRI scan images and family disease histories may be brought together for use in a single model.

Conversely, fusing information as the ultimate step, in which individual classification decisions made by each information source are combined, has traditionally been termed decision fusion.^4^^,^^5^^,^^16^^,^^17^ Voting is a familiar example of decision fusion, both in democratic elections, and in the fail-safe, redundant computer control systems used in nuclear plants^18^ and spacecraft. We hereafter refer to all fusion events that occur after any given mathematical model has assigned a predicted outcome, e.g., a class (such as case vs. control) or an expected ordinal grouping/quantile, for a target variable as decision fusion.

The third category, system fusion, refers to all fusion events that occur between the final training/fitting and cross-validation of mature mathematical models and the conversion of these fitted models to a predicted outcome, e.g., a class such as case vs. control or an expected value (e.g., for a dependent variable in a regression-type analysis).

System fusion approaches have the potential to be more powerful and more flexible than approaches in the other fusion categories. Because system fusion fuses information after the primary modeling step, the individual models may be optimally chosen and parameterized for the specific data types (e.g., continuous vs. ordinal vs. categorical) and distributions encountered, and may be chosen to balance other factors, such as mismatches in the number of variables for a given data source (e.g., weighting five critical clinical variables vs. five million gene alleles). Unlike decision fusion, system fusion approaches may incorporate information about the certainty or confidence of the initial sources (e.g., in the shape of score/probability distributions) that may beneficially influence the fusion result, for instance by giving more weight to sources with more informative or better calibrated score distributions. Our initial paper introducing the DIRAC (diversity of ranks and accuracy) framework contained a review of some current work in this area.^19^

Specifically, system fusion retains information about both the general accuracy of the specific system as well as observation-specific estimates of confidence implied by the location of a given observation in a system’s distribution. In addition, and again in contrast to decision fusion, system fusion approaches similarly retain information about ***diversity*** between the two initial scoring or ranking systems,^12^ both the overall differences between the two systems (e.g., as Spearman rank correlation) and observation-specific contributions to the overall ***diversity*** between the two initial scoring/ranking systems. We documented the importance of these features in the original development of DIRAC for fusion of binary classifiers. To complement the specificity of the many information fusion techniques in this category, we believe that an overall study of system fusion at as general a level as possible is a currently overlooked, but highly worthwhile undertaking.

The advantages noted above would seem to make system fusion the obvious choice for any analysis involving information fusion, but system fusion also has an important weakness, in that, even in well-defined context, it is very difficult (if not impossible) to predict whether a given fusion will or will not be successful (i.e., produce a system that is more accurate than the individual components from which it was constructed). Furthermore, in contrast to decision fusion (i.e., voting, Borda,^1^ Cordorcet^2^, etc.), system fusion has resisted a systematic explication. Worse, the no-free-lunch theorems^20^^,^^21^^,^^22^ predict that the outcome of system fusion is provably impossible to predict in the absence of context.^19^

It has been recognized that fusions are more likely to be successful when the input systems are “good enough” (generalized as “***accuracy***”) to contribute some useful information and “different enough” (generalized as “***diversity***”) such that each system brings novel information.^3^^,^^5^^,^^6^^,^^23^^,^^24^^,^^25^^,^^26^ Unfortunately, despite recognition of this intuitive idea, and the research effort devoted to exploring specific system fusion approaches, quantitative understanding of the relationship between initial accuracy, diversity, and fusion accuracy in the general case has remained elusive.^26^^,^^27^^,^^28^

Our previous research into information fusion, and specifically into system fusion, focused on identifying a method to robustly/rigorously estimate in advance the performance of the fusion of a pair of predictors of known accuracy with respect to binary classification, a problem central to our biological research (i.e., classifying case vs. control). As expected given the relevant literature, we saw that sometimes fusion would improve overall performance, and the averaged predictor would outperform the better of the inputs, and sometimes it would make it worse, with the averaged predictor not improving.

We previously^19^ approached the binary classification problem so as to transcend domain dependence by defining “system” as broadly as possible. Specifically, a “scoring system” was defined to be anything at all that assigns numerical scores to a set of samples. These could represent simple, unprocessed measurements, such as the concentration of glucose in a blood sample, or the probabilistic output of a Bayesian model, or the set of scores assigned by a convolutional neural network. We fused pairs of these scoring systems by simply averaging the scaled scores given to each sample by both scoring systems, and we tested to see whether the fused system induced a better separation of the two classes than either of the pair of input systems.

In this specific context, a strong and consistent relationship was observed^19^ between the individual accuracy of the two input scoring systems (expressed using the area under the receiver operating characteristic curve [AUROC]), the diversity between the two scoring systems (expressed using Pearson correlation [PC]), and the accuracy of the single output scoring system resulting from their fusion. This relationship was robust against wide variation in the score distributions of the two target classes, holding in synthetic data (chosen to exercise our assumptions), and also in a real-world biological/epidemiological dataset. Further study demonstrated that the relationship was even stronger when considering only the relative sample rankings obtained by first converting both initial scoring systems to ranking systems. We refer to this unified formulation of system fusion as the DIRAC framework, and to the specific values of AUROC and the Pearson or Spearman rank correlation that predict the output accuracy of a pairwise system fusion as the DIRAC criteria.

These results immediately raised several deeper questions. How dependent were our results on the specific metrics that we decided to use for our ***accuracy*** and ***diversity*** measurements? Why did scoring system fusions resemble ranking systems fusions with additional noise? Was there a specific mathematical reason/structure underlying our observations? To what extent can our findings generalize to all areas and aspects of information fusion? Here we extend the DIRAC framework and answer these questions.

## Results

One goal of the work presented here is to establish a solid theoretical foundation able to explain the empirical results observed in our previous work. The binary classification problem addressed in our first report^19^ was amenable to direct empirical attack, a factor that allowed us to firmly establish the quantitative roles for ***diversity*** and ***accuracy*** in binary fusion for classification. However, understanding binary classification in terms of sample rankings directly is complicated by the many-to-one mapping between sample rankings and classification accuracy; any two rankings that induce the same distribution of class 1/class 2 (C_1_, C_2_) labels are equivalently accurate. This made the binary classification problem described in our previous study much less amenable to theoretical explanation.

**Figure 1:**
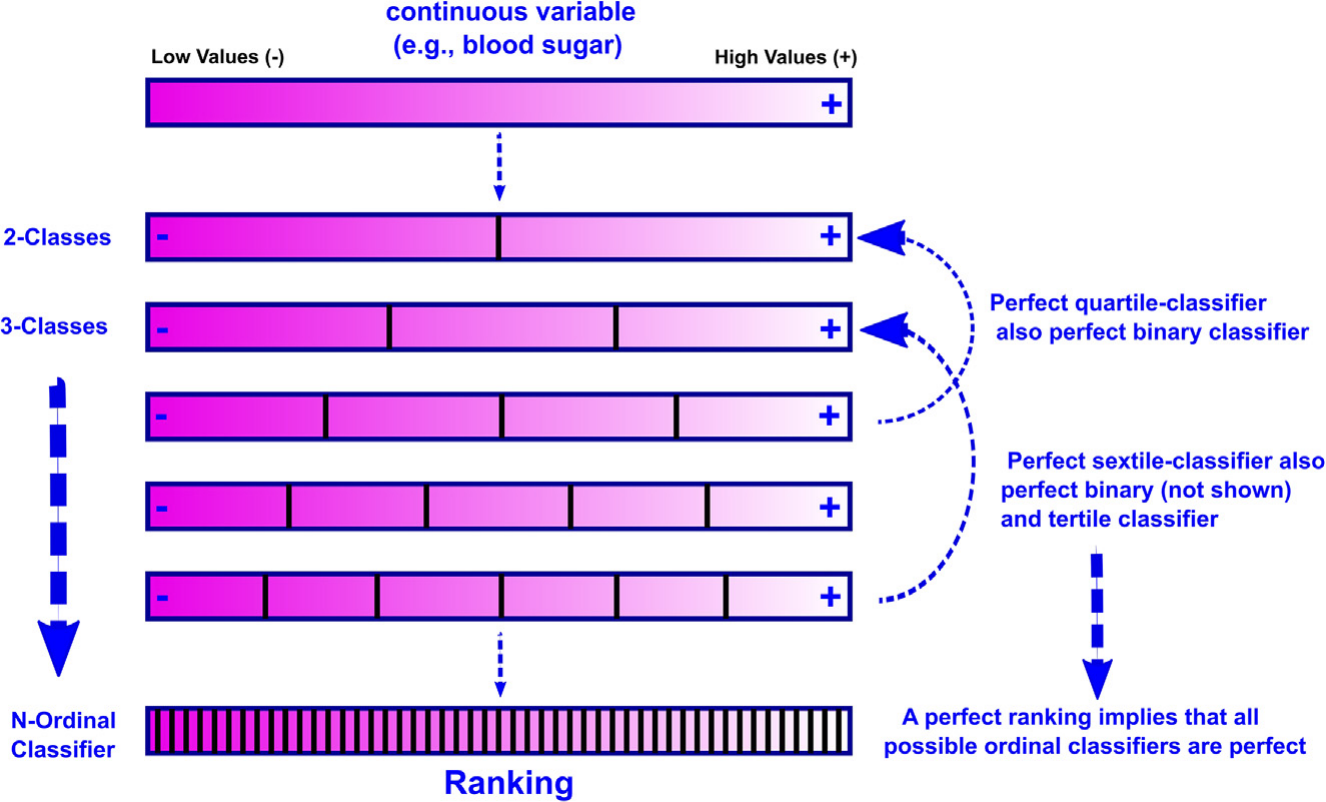
The ordinal ranking continuum from binary classification to rank approximation This schematic shows the sequential breakdown of a continuous distribution into an increasing number of quantiles ranging from two (binary classification, top row) to N (full ranking, bottom row). Moving from top to bottom, the total number of permutations (possible partial rankings) within each quantile decreases, until there is only one element per quantile, yielding the single full ranking. Any perfect ordinal classifier (that correctly orders its component quantiles) also inherently perfectly classifies its “factor” quantiles (i.e., a perfect sextile classifier is both a perfect median and tertile classifier). With a full, correct ranking, all other quantiles are correctly ordered implicitly.

Recognition of this limitation prompted us to modify the DIRAC framework such that the target is a single ranking, with the distance/similarity to this single target ranking serving as the objective to minimize/maximize (respectively) in pairwise fusion. We now seek to use fusion to optimally approximate a single target ranking in place of the binary classification used as an objective in our previous study. As shown in Figure 1, binary classification and rank approximation may be considered as occupying the two extremes on a continuum of ordinal classifications. We use the term “rank approximation” to refer to the extreme N-quantile form ordinal classification in which each observation is given its own, sequentially ordered class.

To study system-level fusion for rank approximation, we initially chose to build directly on the concepts and approaches that we developed previously for binary classification. Specifically, we again began by using simulated data and then validated findings with real-world examples. By initially characterizing fusion between simulated ranking systems, we could determine the relationship between the characteristics of the input systems and the outcome of their fusion without confounding by domain-specific factors including data ascertainment errors and/or scoring errors (such as incorrect labeling). Using these simulations, we exhaustively explored simple averaging of pairs of ranking systems, to determine whether there were conditions in which this average would consistently outperform the better system of the pair. This is pairwise system fusion for rank approximation in its most basic instantiation. As in our earlier reports,^12^^,^^19^ we use the terms “system” and “scoring system” to represent anything that first gives a single numerical assignment to every sample in a population of samples (e.g., analytical test, algorithm, mathematical model). We denote “scoring systems” as “SS_A_,” “SS_B_,” etc., and scale their output from 0 to 1. We use the term “ranking system” to represent anything that ranks (i.e., values from 1 to N) every sample (i.e., observation) in a population of samples. We denote ranking systems, the focus of the current work, as “RS_A_,” “RS_B_,” etc. For the purpose of the work presented here, we break all ties at random, but we recognize and are working on the problem of tied rankings (Sniatynski et al., work in progress). For both scoring and ranking systems, the synthetic data can be interpreted as the score or relative rank in a population of samples evaluated by a single scoring/ranking system. As such, the values reported in SS_A_ reflect the value of a variable of interest independent of other samples in the study (aside from scaling); the values reported in RS_A_ reflect the rankings of the samples in the study when sorted by the scores given by SS_A_. We note that these scoring systems may equivalently reflect diverse underlying processes as complex as fitted ensemble classifiers, or as simple as single clinical measurements, such as fasting blood glucose levels. Given our previous results, we focused on the rankings determined by our input scoring systems rather than the scores, and our objective was to determine the conditions in which a fused ranking system proved more accurate (measured using the Spearman rank correlation) than either of the progenitor ranking systems.

As before, we validated observations using a real-world dataset drawn from the same medical imaging dataset used in our previous paper; this is the Multiethnic Cohort Adiposity Phenotype Study (MEC-APS),^29^ a study of adiposity phenotypes in men and women from five ethnic groups, specifically the initial 1,000 subjects recruited (533 women) consisting of approximately equal numbers of Japanese-Americans, African-Americans, Latino(a)s, Native Hawaiians, and Caucasians. In MEC-APS, body fat distributions were determined using both DXA (dual-energy X-ray absorption) and MRI. We tested DIRAC’s real-world applicability by systematically assessing its ability to predict whether fusing two DXA metrics improved the prediction of a given MRI measurement (defined as ground truth for these purposes) compared with the predictions of either DXA metric alone. Independent of specific biological outcome(s), this experiment tested the mathematical question of whether our simulations reflect real-world performance. We first converted 31 different DXA measurements into ranking systems (assigning ranks on the basis of the magnitude of the measurements), and for each of these we calculated Spearman rank correlation against 39 different MRI measurement targets (variables are listed in Data S1 and S2). We then carried out pairwise system fusion on all possible pairings of DXA measurements, in a manner identical to our simulated data experiments, calculating the change in Spearman rank correlation of the fused predictor (ΔSR_RF[AB]_) against 39 MRI measurement targets. This gives 37,479 fusions plotted in the figures, i.e., DXA (N = 31 variables) × DXA (N = 31 variables) × MRI (N = 39 variables). This number includes self-fusions (N = 1,209, i.e., 31 × 39), and all fusions are double plotted (i.e., each DXA system on the x axis and again on the y axis) for visual clarity. There are 18,135 unique fusions (i.e., [31 × 30 × 39]/2) shown.

### Part I: Rank approximation: DIRAC evaluates fusions of ranking systems.

Single ranking systems are a potentially useful prediction target, and the use of a single, specific ranking system as a target (hereafter RS_T_) greatly simplifies the representation of pairwise system fusion by allowing the accuracy of the two input systems, the diversity between them, and the accuracy of the resulting fused system to all be described using a single metric (i.e., the Spearman rank correlation).

**Figure 2:**
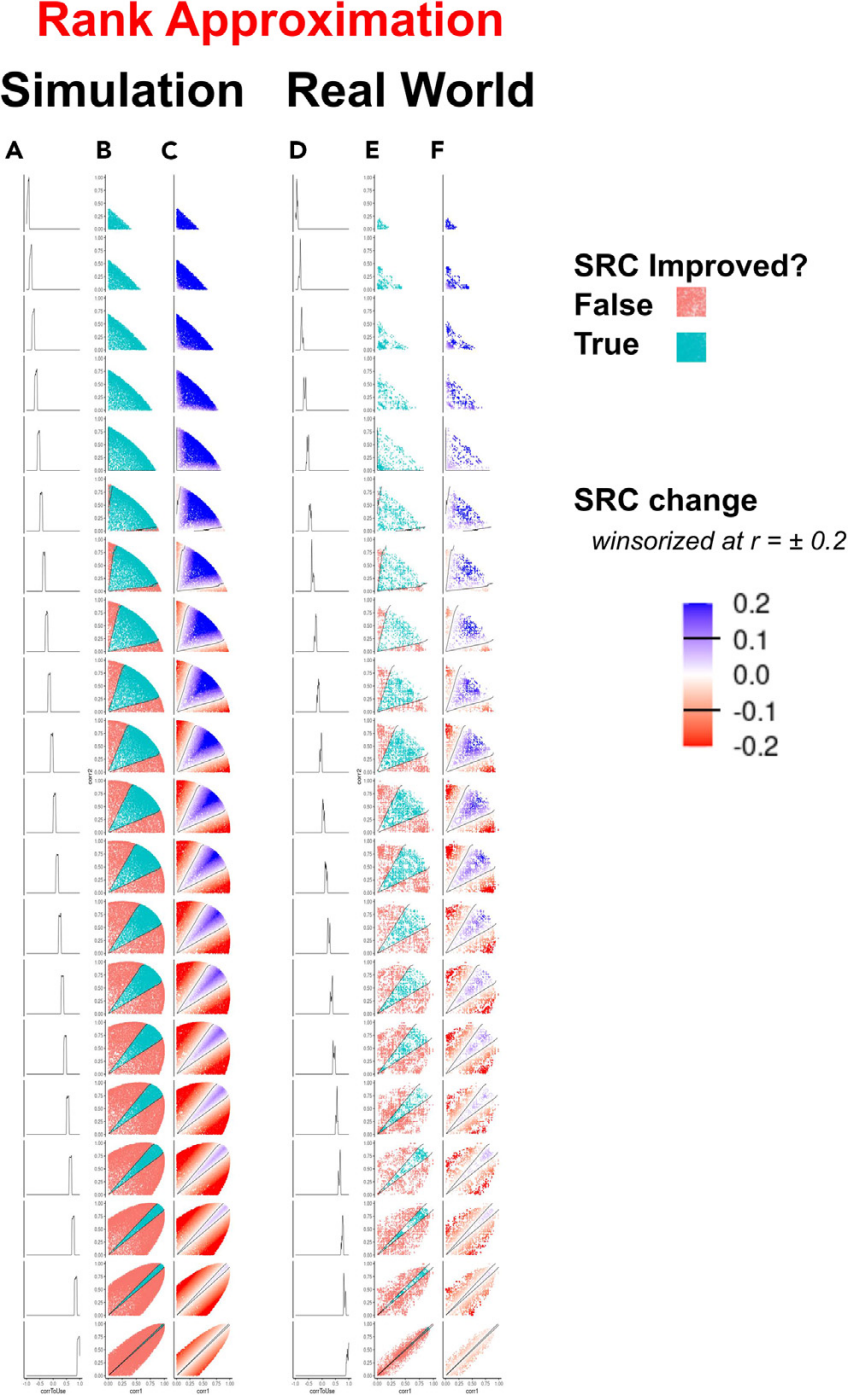
Outcomes of continuous rank fusions are predictable Columns A–C show simulated pairwise system fusion for simulated rank data (generated as described in the experimental procedures). SR is used to measure the similarity of RS_A_ to RS_T_ (x axis) and the similarity of RS_B_ to the RS_T_ (y axis). Each subpanel represents an SR range between RS_A_ and RS_B_ and each row’s range is 0.1 units in width, with the most anticorrelated (−1.0) at the top and the most correlated (1.0) at the bottom. Column A shows a density plot, representing the RS_A_ and RS_B_ SRs found in the corresponding row. Column B points are colored blue if the fusion increased in accuracy over SR_M_ (i.e., ΔSR_RF[AB]_ > 0) and red if it did not (i.e., ΔSR_RF[AB]_ ≤ 0). Column C shows the quantitative increase or decrease in accuracy of the fused system. LOWESS (locally weighted scatterplot smoothing) curves were fit to the simulated rank fusion training data as described previously^19^ and in the experimental procedures. LOWESS curves are generated from data stratified by correlation into 20 bins as before (*r* = −1 to 1, top to bottom, with 0.1 unit intervals) and are superimposed on their cognate data. Each point represents one fusion and its outcome (i.e., two simulated variables and a simulated target). Columns D–F show equivalent, pairwise system fusions using real-world data. Real-world data are identical to those used previously,^19^ but these data are first converted from scores to ranking systems. Briefly, this plot examines whether fusing pairs of DXA measures increases the accuracy of our prediction of a specific MRI measurement. Plots are organized similarly to the simulated data in columns A–C, and columns E and F feature the LOWESS curves from the simulated data (described previously) superimposed. Each point represents one fusion and its outcome (i.e., two DXA variables and a single MRI target). Continuous fusion panels (columns C and F) are winsorized at SR ± 0.20 for visual clarity. Equivalent panels without winsorization and winsorized at ±0.1 and ±0.05 are provided in supplemental information (Figures S1–S3). To make the plots symmetrical and improve visual clarity, each fusion is plotted twice (i.e., once with each system as RS_A_ and again as RS_B_).

We modified the DIRAC simulation/fusion code used in our previous work to operate using a single ranking as the target and repeated the pairwise fusion experiments performed previously. This entailed systematically and comprehensively exploring the effects of ***accuracy*** and ***diversity*** on pairwise fusion performance by creating a large pool of simulated ranking systems (see parameters in experimental procedures) that spanned the range of system accuracies (from 0 ≤ SR ≤ 1 with respect to target) and inter-system diversities. Pairs of ranking systems (RS_A_, RS_B_) were then fused by averaging. We refer to the Spearman rank correlations of the rank fusion RS_A_ and RS_B_ with the target RS_T_ as SR_A_ and SR_B_, respectively, and SR_MAX_ as the Spearman rank correlations of the superior input classifier (i.e., max[SR_A_,SR_B_]). The Spearman rank correlations of the fused system (hereafter, SR_RF[AB]_ for the Spearman rank of the rank fusion of RS_A_ and RS_B_) was measured and compared with SR_MAX_, specifically, ΔSR_RF[AB]_ = SR_RF[AB]_ − SR_MAX_. This quantity is the change in Spearman rank correlation of the fused system (either an increase or a decrease) compared with the more accurate member of the unfused pair. Repeating this pairwise fusion process across the large pool of ranking systems allowed us to explore how ***accuracy*** and ***diversity*** influence the SR_RF[AB]_ of the resulting ranking systems and the ΔSR_RF[AB]_. As in the case of system fusion for binary classification in our previous report,^19^ a visually striking result emerged when the improvement of the fused classifier systems (i.e., ΔSR_RF[AB]_ > 0; presented as binary true/false) was plotted as a function of the two input SRs (SR_A_ and SR_B_; Figure 2). The scatterplot coloring reveals two distinct regions: one central region in which ΔSR_RF[AB]_ > 0 and a peripheral region in which ΔSR_RF[AB]_ ≤ 0). (We note that the apparent classification noise directly adjacent to the boundary in the qualitative plots is due to the thickness of the correlation slice visualized, as in Sniatynski et al.^19^; such points change by minuscule amounts and are visualized as white in the quantitative panels, Figures 2C and 2F.) The results in Figure 2 show that a very similar relationship exists among input ***accuracy***, ***diversity***, and fusion accuracy in the rank approximation context as in the binary classification context. This was initially shown in the simulated data (Figure 2, left panels). We then tested that the relationships visible here in simulated data are reflected in real-world datasets by modeling the boundary between positive and negative fusions in the simulated data using locally weighted scatterplot smoothing (LOWESS) curves, as done initially for binary classification fusion in our previous study, and superimposing these curves on the real data plots (Figure 2, right panels). The simulation-based LOWESS curves describe the real-world fusion boundaries, revealing that the relationship among input ***accuracy***, ***diversity***, and fusion accuracy is identical in both cases. This work empirically extends the binary classification studies in our first report to rank approximation.

### Part II: Geometric framework

The simplified representation and results above point the way to a concise geometric formulation of pairwise system fusion that differs considerably from previously published geometric approaches.^30^^,^^31^ Using this formulation, one can derive the DIRAC relationships observed among input ***accuracy***, ***diversity***, and fused system accuracy, which had previously only been empirically observed and modeled. As introduced above, the extension of the DIRAC framework to rank approximation problems results in a simplified representation of pairwise system fusion, where both input systems and the target are rankings, and ***accuracy*** of two input systems and the ***diversity*** between them are, for the moment, represented using the Spearman rank correlation.

**Figure 3:**
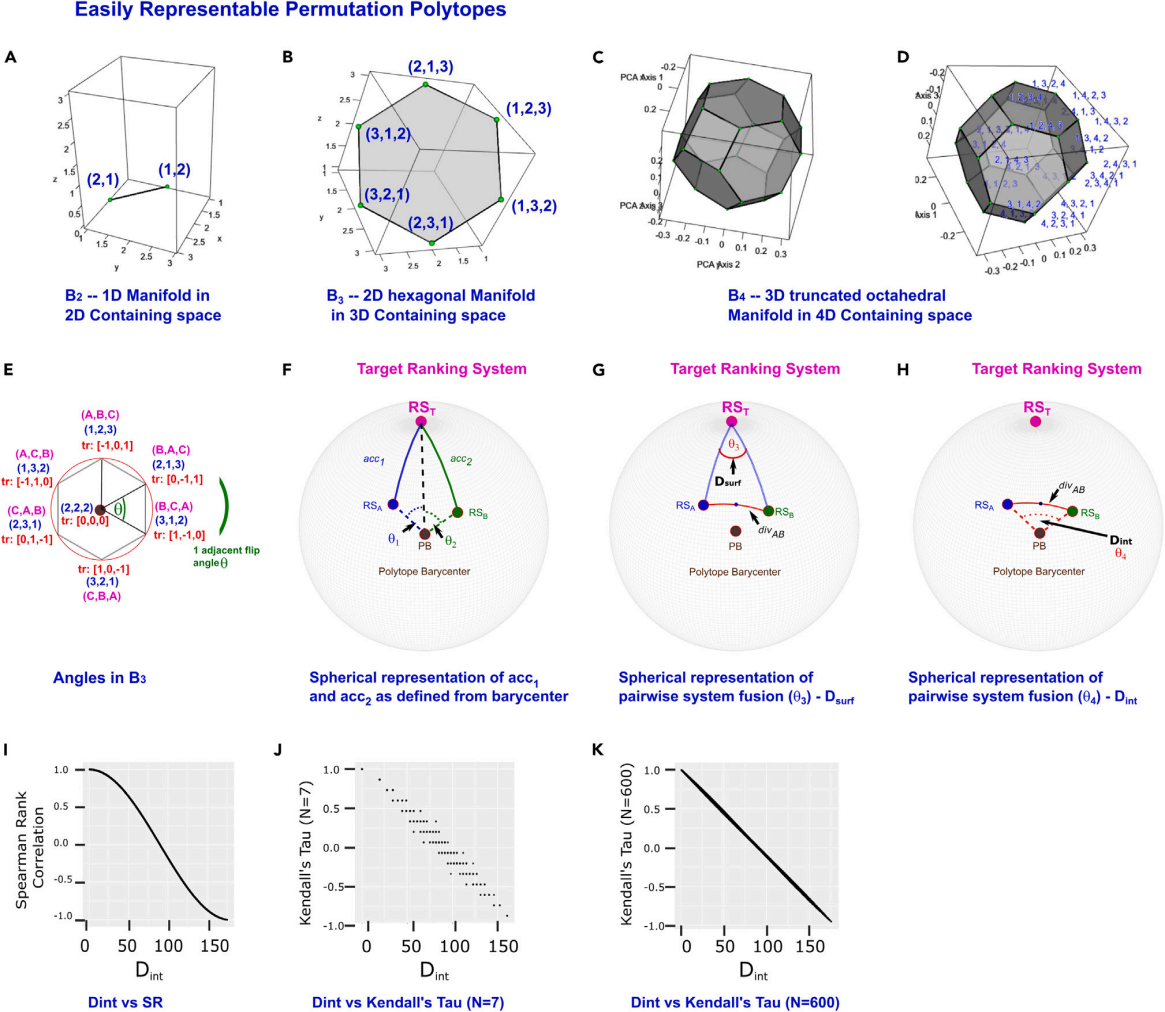
Schematics showing geometric instantiation of DIRAC (A–E) The three permutahedra that are representable within three dimensions without projection and loss of information. These are the line (S_2_, A), the hexagon (S_3_, B), and the truncated octahedron (S_4_, C and D). The truncated octahedron is shown with and without coordinate labels for visual clarity. Interactive versions of these structures are provided in the supplemental information (Data S3 and Data S4). The vertices of these polytopes correspond to a particular ranking: the location of a vertex in Euclidean space is obtained by treating the ranking as a coordinate. Note that for the line, the rankings of the endpoints correspond to Euclidean coordinates in the plane, and for the hexagon, the rankings at the six vertices correspond to Euclidean coordinates in the three-dimensional space in which the hexagon is embedded. The four-dimensional coordinates corresponding to the ranking vertices of the truncated octahedron cannot be directly visualized in this way in our 3D world, but the above demonstrates the general properties of all of these convex N-1 dimensional manifolds. The use of these polytopes in Euclidean space is simplified by the translation of the barycenter of the permutahedron to the traditional Cartesian origin [i.e., (0, 0,…0)]. We hereafter denote the “raw” coordinates as (X, Y, Z…), and we denote the translated coordinates as tr: (X, Y, Z…). This is shown in (E) using the hexagonal permutahedron S_3_. Here we show the observation’s trivial designation (here, [A, B, C]), the rank of each of its features (here ranging from 1 to 3) and the translated rank of each of its features (here ranging from −1 to 1). From the central Euclidean/Cartesian origin point of tr: (0, 0, 0), it is evident that discretized angles (e.g., θ) can be used to represent, as an actual distance (i.e., d_1_ in E), one or more adjacent transpositions between neighboring rankings. This approach generalizes to higher dimensions. (F–H) The geometry of pairwise system fusion featuring two candidate ranking systems to fuse (RS_A_ and RS_B_), the target ranking system RS_T_) here represented as being at the “North Pole” of a sphere), and the polytope barycenter (PB)/origin at the center of the polytope. These form the set of four points necessary to define a three-dimensional sphere, which allows ***accuracy*** and ***diversity*** to be represented geometrically: the ***accuracy*** of a given ranking system is the angular distance away from the origin-target axis (similar to latitude in the representation shown where the target is the “North Pole”), and the ***diversity*** between the two ranking systems is representable by either the rotation needed around the origin-target axis separating the two, here represented by D_surf_ (analogous to longitude in the representation shown) or by the angular distance separating the two candidate systems directly (D_int_). For clarity, we note that RS_A_, RS_B_, and RS_T_ are all points that lie on the surface of the 3-sphere, whereas PB is its barycenter. Likewise, note that angle θ_3_ thus lies on the surface of the 3-sphere and is defined by the intersection of two arcs, but θ_1_, θ_2_, and θ_4_ all i.e., within the interior of the 3-sphere and are defined with a vertex at the translated barycenter (i.e., tr: [0, 0, 0]). (F) highlights the ***accuracy*** measures of RS_A_ and RS_B_. Distances between these systems and the RS_T_ are captured by the arcs RS_A_-RS_T_ and RS_B_-RS_T_, denoted *acc*_1_ and *acc*_2_ (when we are referring to the surface distance) and the angles θ_1_ and θ_2_ when we are referring to the interior angles that (respectively) subtend these arcs. Note that, for a unit sphere the angles in radians are the lengths of the surface arcs. (G) D_surf_, defined by the surface angle θ_3_ that describes the rotation at the target point (i.e., “longitude”) separating RS_A_ and RS_B_. whose surface distance we will define as div_AB_. We note div_AB_ lies on the geodesic/great circle route. (H) D_int_, defined by the interior angle θ_4_ that subtends the spherical surface arc between SS_A_ and SS_B_ and thus has the surface distance div_AB_. (I–K) Relation of D_int_ to SR and Kendall’s tau (KT).

A ranking system consists of the ordering into which the corresponding scoring system would arrange the samples in a dataset, if they were sorted by this score (i.e., RS_A_ comprises the cognate ranks from SS_A_). Although this transformation from scores to ranks is often treated simply as a lossy, quantized representation of the score information, ranking systems may also be described from the viewpoint of the field of combinatorics, as permutations of the set of natural numbers with cardinality N, with N corresponding to the total number of samples in the dataset. This set of possible ranking permutations is finite for a given N, and from a group theoretic perspective this set forms a symmetric group of order N (denoted S_N_). Permutations such as these rankings, and the transformations connecting them, are most commonly represented within a bubble sort Cayley graph (denoted B_N_) when there are no tied rankings^32^^,^^33^ and within the Kemeny rank space (denoted H_N_) when there are tied rankings.^34^^,^^35^^,^^36^^,^^37^^,^^38^ In these symmetric structures, every possible full ranking (i.e., without ties) is associated with a vertex in the cognate polytope. These permutation polytopes may also be represented in Euclidean space. When these are extended to the Euclidean space, the symmetric groups feature regular geometric structure which forms a convex polytope of dimension N − 1, embedded as a manifold within the containing N-dimensional space (e.g., B_3_ is a 2-dimensional hexagon embedded in a three-dimensional space).^32^^,^^33^^,^^36^^,^^37^^,^^39^^,^^40^ For example, Figures 3A–3C show the three permutation polytopes (also referred to as permutahedra) that may be readily displayed within three dimensions; these correspond to the bubble sort Cayley graphs containing two, three, and four elements denoted B_2_, B_3_, and B_4_, respectively. These three permutation polytopes are the line, the hexagon, and the truncated octahedron. As is directly visible in the B_2_ and B_3_ case, we can treat these permutahedra as being embedded in Euclidean space, with the rankings of each vertex mapping to the Cartesian coordinates locating them in this space (Figures 3A, 3B, 3D, and 3E). The edges of the polytope correspond to the adjacent transpositions that link neighboring pairs of rankings in bubble sort Cayley graph or Kemeny rank space, such that any route along these edges between vertices P_1_ and P_2_ corresponds to the sequence of such adjacent transpositions necessary to transform ranking P_1_ into ranking P_2_. Importantly, we can draw a direct line between any two vertices in the permutahedron, and derive the coordinates of any point on this line by weighting the endpoints. Because the permutahedron is a convex hull, all such points are located inside it, and will not represent full rankings (i.e., a permutation of the natural numbers from 1 to N).^41^ However, transforming the interior point coordinates into a full ranking will map this interior point to the closest full ranking on the permutahedron surface (note that several such surface points may be equidistant to this interior point. This is the result when the ranking system fusion provides tied rankings).

Because the rank approximation formulation of pairwise system fusion described in part I represents both input systems and the target as rankings, and therefore as permutahedron vertices, we propose that the DIRAC relationships observed among input system ***accuracy***, ***diversity***, and fusion accuracy may be mathematically derived from the geometry of the corresponding permutahedron.

Initial translation of the permutahedron simplifies the required geometric analysis. Specifically, an angle-based representation of ***accuracy*** and ***diversity*** requires an origin point located at the barycenter of the ranking system vertices. The angle between the vectors from this origin to the ranking system vertices in question represents the distance across the surface between any two rankings. For convenience, we translate the entire permutahedron structure so that its central point (barycenter) is located at the Cartesian origin ([0, 0,⋯, 0]), which is achieved by subtracting the mean rank (common to each possible ranking in a dataset of size N) from each element/coordinate of each ranking; for example, the ranking ([3, 4, 1, 2]) would become ([0.5, 1.5, −1.5, −0.5]) when translated. For clarity, we hereafter refer to the translated coordinates as tr: [X,Y,Z, …], i.e., [3, 4, 1, 2] would become tr: [0.5, 1.5, −1.5, −0.5]. Figure 3E shows the translated version of B_3_ including both untranslated and translated coordinates (in blue and in red, respectively). It is evident from this figure that the angle at the origin between ranking system vectors directly reflects the distance traveled across the surface of the (N − 1) sphere, and that this distance is in turn proportional to the number of adjacent transpositions necessary to transform one ranking system into another one. Being permutations of the set of natural numbers {1, 2,…, N}, each ranking system is located the same distance away from the origin, so this geometric intuition scales to any dimension, and the spherical approximation allows the angle between any two ranking systems to be calculated as the inverse cosine of the dot product of the translated ranking system vectors (see experimental procedures).

Within this geometric framework, pairwise system fusion may be described using tools of spherical geometry (see Figures 3F–3H) by using the four points minimally necessary and sufficient for the definition of a three-dimensional sphere (hereafter, 3-sphere); these are the target point (or target ranking system, abbreviated as RS_T_ in Figures 3F–3H), the two input systems to be fused, RS_A_ and RS_B_, and the Cartesian origin point (the barycenter of the permutahedron after translation, i.e., tr: [0, 0, 0], abbreviated as PB in Figures 3F–3H). By treating the RS_T_ as a reference (like a spherical “pole”), we can locate the candidate system points RS_A_ and RS_B_ using the equivalents of “latitude” and “longitude” on the 3-sphere. It is sufficient to measure the angular distance separating the two candidate system points from each other (i.e., their diversity, arc RS_A_-RS_B_ which is of length *div*_AB_ in Figures 3G and 3H), along with the angular distance separating each of these from the target point (i.e., their accuracy, arcs RS_A_-RS_T_ and RS_B_-RS_T_ which are of lengths *acc*_1_ and *acc*_2_ respectively in Figure 3F). Using this geometric representation, pairwise system fusion may now be readily visualized as in Figures 3F–3H, with the relationships between the points and the angles amenable to analysis using spherical trigonometric identities.

We refer to the angles at the barycenter as interior angles, and to those on the spherical surface as surface angles. The interior angles RS_T_-PB-RS_A_ and RS_T_-PB-RS_B_ (θ_1_ and θ_2_ in Figure 3F, respectively) define the arcs RS_A_-RS_T_ and RS_B_-RS_T_. As noted above, the lengths of these arcs, *acc*_1_ and *acc*_2_, are the respective accuracies of RS_A_ and RS_B_. The surface angle formed by the arcs that run from the RS_A_ or RS_B_ to the RS_T_ with its vertex at the RS_T_ (i.e., RS_A_-RS_T_-RS_B_) is labeled θ_3_ in Figure 3F and termed D_surf_. D_surf_ conceptually represents the ***diversity*** of the ranking systems from the point of view of the target point RS_T_. D_surf_ is analogous to the within-class correlation described in our previous study^19^ in that, for a given pair of input systems, it is independent of their relative accuracies; i.e., while one needs the RS_T_ to define D_surf_, the value of the angle θ_3_ is independent of the accuracy of either input system. Finally, the interior angle at the polytope barycenter between the pair of ranking systems (RS_A_-PB-RS_B_) is labeled θ_4_ in Figure 3H and termed D_int_. D_int_ represents the diversity of the ranking systems from the point of view of the PB. D_int_ is thus fully independent of the RS_T_, but there are constraints on the possible combinations of the accuracies of the input systems and the D_int_ between them (e.g., two highly accurate models cannot also have a large D_int_ between them). Note that, given the accuracies of both input systems, D_surf_ can be used to calculate the length of the arc subtended by D_int_.; thus, as would be expected given the added context of the RS_T_, both D_surf_ and D_int_ can be used to reach identical predictions for the outcome of a given fusion (data not shown). We note that D_int_ is related to other relevant correlation metrics, Spearman rank correlation, and Kendall’s tau (KT; Figures 3I–3K).

Pairwise mean fusion creates a new ranking system located as close as possible to the mid-point of the shortest path distance across the surface of the 3-sphere connecting the input system points RS_A_ and RS_B_ (i.e., *div*_AB_); in spherical geometry such a shortest path is referred to as a “great circle” or “geodesic.” Because the vertices of the underlying permutahedron are discretely located, we note the RS_A_-RS_B_ geodesic midpoint need not be precisely located at a permutahedron vertex. The RS_A_-RS_B_ geodesic midpoint will instead likely be located at a “degenerate” ranking coordinate: a valid point in Euclidean space on the approximating sphere, but not a full ranking and thus not a valid permutation in the corresponding Cayley graph. This happens because the pairwise mean fusion of two ranking systems may produce ties in the fused system; this degenerate ranking system is equidistant from two or more vertices on the convex hull of the underlying permutahedron. These equidistant vertices on the convex hull represent all possible ways of breaking the tie(s).

### Part III: Geometric ***accuracy*** and ***diversity***

**Figure 4:**
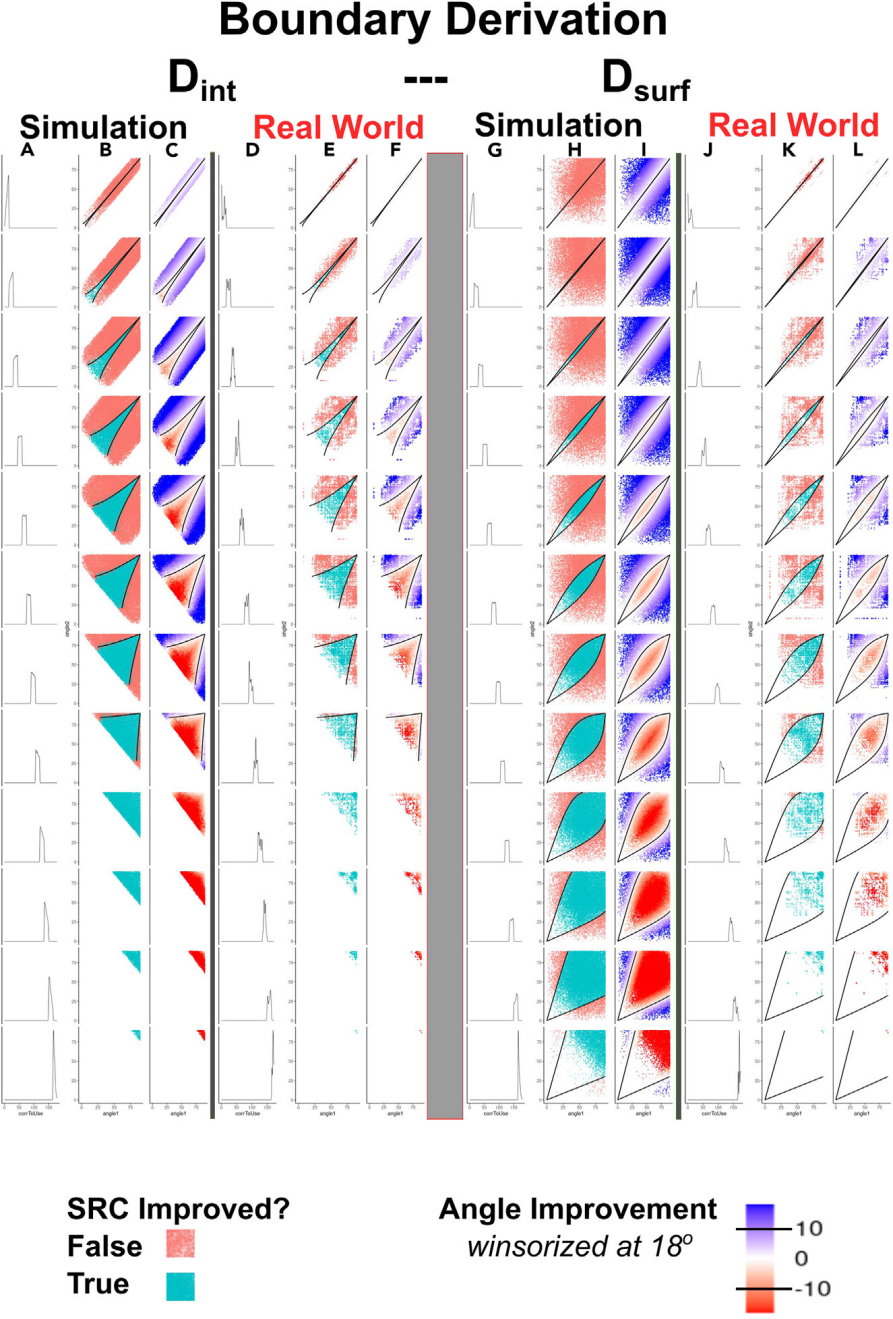
The geometric instantiation of DIRAC directly provides boundaries for rank approximation fusions Columns A–F show fusion boundary determination and validity using the internal angle D_int_; columns G–L show fusion boundary determination and validity using the surface angle, D_surf_. The general layout follows Figure 2: in each set of six, the first three columns show simulated data, the last three show real data. The leftmost rows of each triplet (A, D, G, and J) show a density plot, with each row representing an angle D_int_ (A and D) or D_surf_ (G and J) range between RS_A_ and RS_B_ that is 20° in width, with most anticorrelated (max of 180°) at the top, and most correlated (minimum of 0°) at the bottom. In the remaining columns, we use the interior angles (θ_1_, θ_2_ in Figure 3F) to measure the similarity of RS_A_ to RS_T_ (x axis), the similarity of RS_B_ to RS_T_ (y axis). In the second figure in each triplet (B, E, H, and K), points are colored blue if the fusion increased in accuracy over SR_MAX_ (i.e., ΔSR_RF[AB]_ > 0) and red if it did not (i.e., ΔSR_RF[AB]_ ≤ 0). The third column in each triplet (C, F, I, and L) shows the quantitative increase or decrease in accuracy of the fused system. We then use the spherical approximation of the geometry of the permutahedron to derive the boundary between ΔSR_RF[AB]_ > 0 and ΔSR_RF[AB]_ < 0, when using D_int_ or D_surf_ as the measure of ***diversity***. Because the sign of ΔSR_RF[AB]_ changes at the boundary, the fusion system point (the ranking system closest to the center of the geodesic connecting RS_A_ and RS_B_) and the higher accuracy input system (by convention RS_A_) must be equidistant to the target. As the diversity between RS_A_ and RS_B_ is fixed, the requirement that the triangle RS_A_-M-RS_T_ (where M is the midpoint between RS_A_ and RS_B_) be isosceles for D_int_ and D_surf_ enables us to use spherical trigonometric identities to determine the corresponding RS_B_-RS_T_ for any given value of RS_A_-RS_T_. To derive the boundary, fix the specific diversity D_int_ or D_surf_ to the desired value, construct/sample a vector of desired RS_A_ accuracies (*acc*_1_ and angle θ_1_), and solve for the corresponding RS_B_ accuracies. These points define the boundary between ΔSR_RF[AB]_ > 0 and ΔSR_RF[AB]_ < 0. We note that these boundaries are accurate for both simulated and real-world data (cf. columns B and C with E and F and columns H and I with K and L). Continuous fusion panels (columns C and F) are winsorized at ±18° (approximately equivalent to a change in SR of 0.05, although this depends on initial SR; see Figure 3I) for visual clarity. Equivalent panels without winsorization and winsorized at 4.5° and 9° are provided in supplemental information (Figures S4–S6). To make the plots symmetrical and improve visual clarity, each fusion is plotted twice (i.e., once with each system as RS_A_ and again as RS_B_).

**Figure 5:**
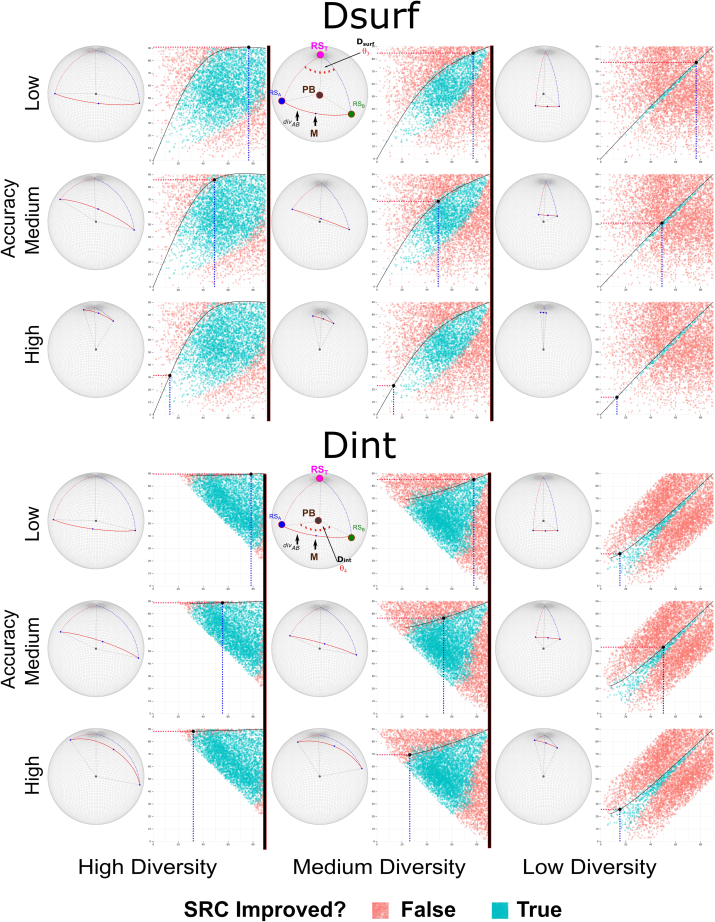
Visualizing the interaction of ***accuracy*** and ***diversity*** in rank approximation fusions As noted in the text and in Figure 4, the geometric formulation of DIRAC enables us to analytically derive the boundary between ΔSR_RF[AB]_ > 0 and ΔSR_RF[AB]_ < 0 for rank approximation by using two different but related geometric formulations of pairwise system ***diversity***: D_int_, and D_surf_. The top set of nine panels uses D_surf_, which defines ***diversity*** as the angle at the target point, between the spherical arcs (geodesics) that connect both candidate systems with the target point (analogous to the North Pole) on the surface of the 3-sphere. Coupling this with the analogous linkage between ***accuracy*** and latitude allows us to represent the interactions of ***accuracy*** and the D_surf_ measure of ***diversity*** in a spherical context that is visually familiar to most people. Reading across the columns from left to right, one moves from high to low diversity: the equivalent of moving from large to small longitude differences (note that to enable direct comparison, ***diversity*** as D_surf_ is fixed for all panels in each column). Reading down the columns one moves from low to high accuracy: the equivalent of moving progressively northward in the spherical analogy that we have been using. The bottom set of nine panels uses D_int_, which defines ***diversity*** as the angle at the barycenter (by analogy equivalent to the center of the earth), between the vectors corresponding to the pair of ranking systems (RS_A_, RS_B_) on the surface of the 3-sphere. Reading across the columns from left to right, one moves from high to low diversity – the equivalent of moving from large to small distances apart on the Earth’s surface (note, to enable direct comparison, ***diversity*** as D_int_ is fixed for all panels in each column). Reading down the columns, one moves from low to high accuracy: the equivalent of moving progressively northward in the spherical analogy. For visual reference, the system and angle notation from Figures 3F–3H is added to the low accuracy/middle diversity panels.

Extended consideration of the geometric framework above now allows us to establish the theoretical underpinnings of system fusion for rank approximation. Specifically, in Part II [above], we developed a geometric formulation of pairwise system fusion from a spherical approximation of the permutahedron structure that arises naturally when dealing with ranking systems. In this formulation, the target and the two input systems may be represented by the three “corners” of a spherical triangle. The length of the sides of this triangle (i.e., *acc*_1_, *acc*_2_, and *div*_AB_), defined by the interior angles introduced above θ_1_, θ_2_, and θ_4_ (D_int_) respectively, represent geometric equivalents of the ***accuracy*** and ***diversity*** measures central to the DIRAC framework. Appropriate visualizations of simulations and of real data (Figure 4) show the results of a similar set of pairwise fusions as that from Figure 2 in part I but that use the angles θ_1_, θ_2_, and θ_4_ (D_int_) in place of the Spearman rank correlations. Figure 4 shows these in the context of the strip plots used above (Figure 2) and in or previous report^19^; Figure 5 shows specific examples highlighted in the context of the polytope geometry and the 3-sphere, as shown in Figure 3. Figure 4 thus emphasizes the global role of ***accuracy*** and ***diversity***; conversely, Figure 5 focuses more on “mechanism,” using specific examples in two 3 × 3 grids (D_surf_ and D_int_) to bring clarity to the relative effects of ***accuracy*** and ***diversity***.

The angles θ_1_, θ_2_, and θ_4_ correspond directly to the three sides of the spherical triangle (*acc*_1_, *acc*_2_, and *div*_AB_, respectively) in Figure 3F, and as the figure demonstrates, representing ***accuracy*** and ***diversity*** using these angles preserves the DIRAC relationships. This approach conveys equivalent information and yields identical results as those generated using SR although they do so in a visually distinct way (Figure 4, left panels); this result is unsurprising, as the direct equivalence in this context between SR and interior angle was demonstrated in Figure 3I.

We posit that, as noted above, surface angle diversity (D_surf_; Figure 3F) represents the rank approximation equivalent of the “within-class correlation” that we developed in our previous study of the fusion characteristics of binary classifier systems; this measure was developed with the specific goal of removing the influence of the difference in accuracies from the measured ***diversity***. In informal terms, D_surf_ represents the diversity between the pair of input ranking systems from the point of view of the target, and D_surf_ is thus an alternative representation of ***diversity*** from D_int_. As D_surf_ corresponds to an angular distance around an axis connecting the translated barycenter (i.e., the Cartesian origin [0, 0,⋯, 0]), to the target point, D_surf_ is, by definition, orthogonal to this axis, and therefore geometrically independent of the accuracy of either of the two input systems. Using this angle to represent ***diversity*** within the DIRAC framework “filters out” any RS_A_-RS_B_ ***diversity*** that originates only from the difference in ***accuracy***. The ability to consider D_surf_ in addition to D_int_ enables complementary domain-specific insights from the interaction of ***diversity*** and ***accuracy*** (see discussion).

Repeating once more the series of pairwise fusions carried out above, but substituting D_surf_ for D_int_ produces Figure 4 (right panels) for simulated data and real data. As in the earlier Figures, these visualizations again show that the DIRAC approach clearly and robustly distinguishes fusions that did improve from fusions that did not improve. Although the appearance of these plots is very different from the D_int_/SR case (e.g., Figure 4, left panels), it is satisfying to note that, because of the accuracy-independence of D_surf_, these plots now strongly resemble the plots from our previous binary classification work, which used the aforementioned within-class correlation as a measure of diversity. The D_surf_ and D_int_ cases are linked via trigonometric identities, and thus yield identical predictions.

With this geometric framework in hand, it is possible to analytically derive the boundary between these regions for the case of rank approximation. Geometrically, the limiting case for fusion improvement with a fixed D_int_ occurs when the more accurate member of the pair (RS_A_ and RS_B_) lies at exactly the same distance from the target point (RS_T_) as the midpoint of the geodesic directly connecting RS_A_ and RS_B_. As explained above, the length of this connecting geodesic (represented by the interior angle D_int_) measures the diversity between the two systems. From the geometry of this limiting case, we see that for any given RS_A_ accuracy value, it is possible to use spherical trigonometric identities to solve for the corresponding RS_B_ accuracy. This RS_A_/RS_B_ pair, by construction, will lie on the boundary that separates pairwise fusions that improved correlation with the target RS_T_ (i.e., ΔSR_RF[AB]_ = SR_RF[AB]_ − SR_MAX[AB]_ > 0 and its angle-based equivalent) from those that produce a lower correlation with the target RS_T_. Holding the D_int_ angle constant at any arbitrarily selected ***diversity*** that we wish allows us to generate a range of RS_A_ and RS_B_ values that map out the entire fusion ΔSR_RF[AB]_ boundary, specific to this fixed diversity value. This derived boundary perfectly separates the ΔSR_RF[AB]_ > 0 from ΔSR_RF[AB]_ < 0 fusions in simulated data, as is shown in detail in Figures 4A–4C. Likewise, it is possible to analytically derive the boundary for a fixed D_surf_, when using the surface angle version of ranking system ***diversity***. Although the calculations are more complicated in this case (see experimental procedures), the limiting boundary case is shown in Figures 4G–4I), and it is again possible to derive the RS_B_ accuracy value that corresponds to a given RS_A_ accuracy value, for any fixed value of D_surf_, using spherical trigonometric identities. The derived D_surf_ boundary case is plotted on top of simulated data in Figures 4H and 4I, where we again see perfect separation between ΔSR_RF[AB]_ > 0 from ΔSR_RF[AB]_ < 0 fusions.

For completeness, we now repeat the analysis performed in part I of this study, using simulated data (Figures 4A–4C and 4G–4I, interior angle and surface angle, respectively) and real-world data (Figures 4D–4F and 4J–4K, interior angle and surface angle, respectively), replacing the LOWESS model-derived boundary curves with the analytically derived curves described above. In all cases, these derived boundaries perfectly separate the regions where the fusion improved in accuracy from the regions where it did not.

The geometric framework developed formalizes our understanding of DIRAC’s ability to predict the outcomes of system fusions for rank approximation. In the next section, we show that this approach can be generalized to establish the theoretical underpinnings of the binary classification findings in our previous report.

### Part IV: The geometry of binary classification

The geometric system fusion framework that we developed in part III, where the target and the two candidate systems are single rankings, and the ***accuracy*** and ***diversity*** are represented using angles, is now sufficiently generalized to be equally applicable to the binary classification case that originally motivated the formulation of the DIRAC framework, and which we described in our previous report.^19^ The principal difficulty in this application is that there are many possible rankings corresponding to a single binary classifier accuracy; any permutation of the rankings belonging to a single class will change the overall ranking (and correspond to a different vertex on the permutahedron), but will not alter its accuracy as a classifier.

When both ***accuracy*** and ***diversity*** are quantified using angles, as in the geometric DIRAC formulation of part III, the only important information is the direction in which each ranking system vector is pointing relative to the RS_T_. The magnitude of the ranking vectors provided no additional information; each ranking is simply a permutation of the same set of natural numbers, and therefore all are equal in length. By considering the permutations (ranking systems) as Euclidean vectors, we can use the same mathematical machinery to address fusions involving tied rankings that we do for full rankings. This, in turn, directly enables us to represent classification tasks, which are essentially tied outcome rankings, within the same geometric framework.

To elaborate, in a binary classification task the single “perfect” target ranking will order all of the class 1 (C_1_) samples before (or after) all of the class 2 (C_2_) samples. With N total samples (and assuming for simplicity and without loss of generality that the classes are balanced), such a situation will involve all C_1_ samples having ranks drawn from the set [1, 2, ⋯, N/2], and C_2_ samples having ranks from the set [N/2 + 1, N/2 + 2, ⋯, N] (or vice versa, respectively). Any randomly generated full ranking that conforms to this requirement will perfectly separate the two classes. (Note that the example ranking sets above are the “natural” rankings, before the translation of the permutahedron to the Cartesian origin.)

We can now link the formalized geometric framework laid out above to the binary classification problem that we previously explored empirically by defining a single target ranking that describes the binary classification task. Although it may seem intuitively impossible to construct a single target vector for this problem that simultaneously points in the same direction as all possible perfect vectors, we instead focus on the recognition that what is needed is a target vector whose maximum dot product will occur with *any and all* rankings that perfectly separate the two classes. The definition of the dot product shows that this is only possible when the target contains only two distinct values, the first of which is repeated for each target index corresponding to C_1_, and the second of which is likewise repeated at each C_2_ index. When this is the case, the aggregate contribution to the dot product from each class will always be the same, regardless of the within-class permutation. We therefore construct the artificial target vector such that the mean of the C_1_ rankings (in the perfectly separated case outlined above) occupies each position corresponding to a C_1_ sample, and likewise for C_2_. The vectors that attain the maximum possible dot product with this artificial vector are then only those that perfectly separate the two classes.

The target in a full ranking situation is a ranking system located at a vertex on the surface of the corresponding permutahedron, but the “artificial” ranking constructed as the target in the case of binary classification is not a proper ranking (being full of ties) and does not exist at a vertex in the corresponding permutahedron. However, it is still possible to conceptualize the vector from the origin (barycenter) to this artificial point as the target; this enables us to use exactly the same angle-based techniques as before to represent pairwise system fusion.

**Figure 6:**
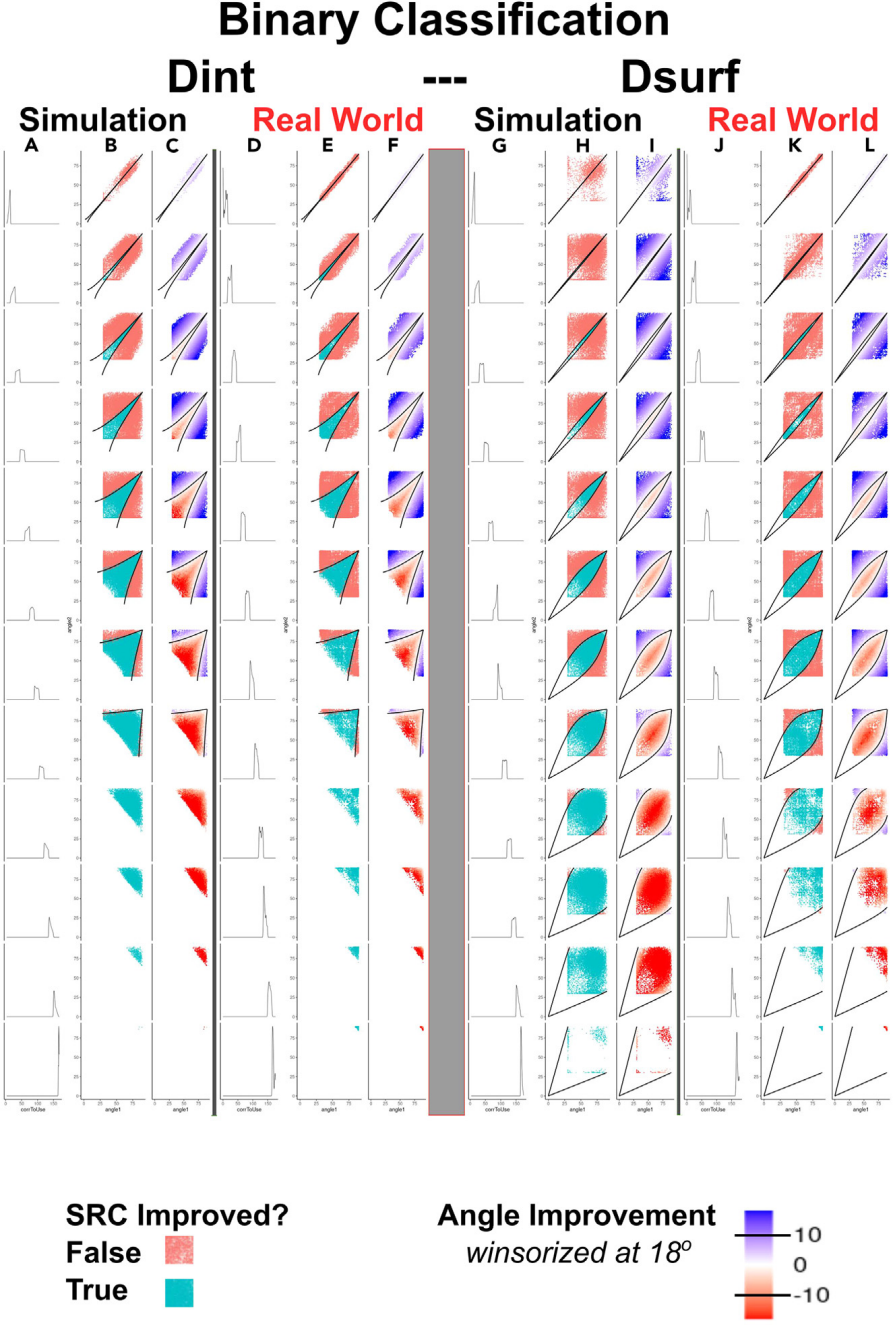
The geometric instantiation of DIRAC directly provides boundaries for binary classification fusions When binary classification is represented using a tie-filled “artificial” target ranking system, the angle between any given ranking system, i.e., RS_A_ or RS_B_, and this target is directly proportional to the AUROC of that system. Binary classification examined as in our previous study^19^ used simulated data to generate LOWESS curves; here, instead, we determine the values directly using the classification-angle approach developed in this section instead of AUROC to measure ***accuracy*** and using D_int_ or D_surf_ to measure ***diversity***. Thus, the figure and legend follow Figure 4 but extends the analysis from full ranking (i.e., the bottom of Figure 1) to binary classification (the top of Figure 1 and the subject of our previous report). Specifically, columns A–F show fusion boundary determination and validity using the internal angle D_int_; columns G–L show fusion boundary determination and validity using the surface angle, D_surf_. In each set of six, the first three columns show simulated data, the last three show real data. The leftmost rows of each triplet (A, D, G, and J) shows a density plot, with each row representing an angle D_int_ (A and D) or D_surf_ (G and J) range between RS_A_ and RS_B_ that is 20° in width, with most anticorrelated (maximum of 180°) at the top, and most correlated (minimum of 0°) at the bottom. In the remaining columns, we use the interior angles (θ_1_, θ_2_ in Figure 3F) to measure the similarity of RS_A_ to RS_T_ on the x axis (Note that RS_T_ here is the tied vector distinguishing class 1 from class 2, and the similarity of SS_B_ to RS_T_ on the y axis.) In the second figure in each triplet (B, E, H, and K), points are colored blue if the fusion increased in accuracy (i.e., ΔSR_RF[AB]_ > 0 or its angle equivalent) and red if it did not (i.e., ΔSR_RF[AB]_ ≤ 0). The third column in each triplet (C, F, I, and L) shows the quantitative increase or decrease in accuracy of the fused system. We then use the spherical approximation of the geometry of the permutahedron to derive the boundary between ΔAUC_RF[AB]_ > 0 and ΔAUC_RF[AB]_ < 0, when using D_int_ or D_surf_ as the measure of ***diversity***. Because the sign of ΔAUC_RF[AB]_ changes at the boundary, the fusion system point (the ranking system closest to the center of the geodesic connecting RS_A_ and RS_B_ and the higher scoring input system must be equidistant to the target. As the diversity between RS_A_ and RS_B_ is fixed, the requirement that the triangle RS_A_-M-RS_T_ (where M is the midpoint between RS_A_ and RS_B_) be isosceles for D_int_ and D_surf_ enables us to use spherical trigonometric identities to determine the corresponding RS_B_-RS_T_ for any given value of RS_A_-RS_T_. To derive the boundary, fix the specific diversity D_int_ or D_surf_ for a given fusion to the desired value, construct/sample a vector of desired RS_A_ accuracies (i.e., *acc*_1_ and angle θ_1_), and solve for the corresponding RS_B_ accuracies. These points define the boundary between ΔAUC_RF[AB]_ > 0 and ΔAUC_RF[AB]_ < 0. We note that these boundaries are accurate for both simulated and real-world data (cf. columns B and C with E and F and columns H and I with K and L). Continuous fusion panels (columns C and F) are winsorized at ±18° (approximately equivalent to a change in SR of 0.05, although this depends on initial SR; see Figure 3I) for visual clarity. Equivalent panels without winsorization and winsorized at 4.5° and 9° are provided in supplemental information (Figures S7–S9). To make the plots symmetrical and improve visual clarity, each fusion is plotted twice (i.e., once with each system as RS_A_ and again as RS_B_).

**Figure 7:**
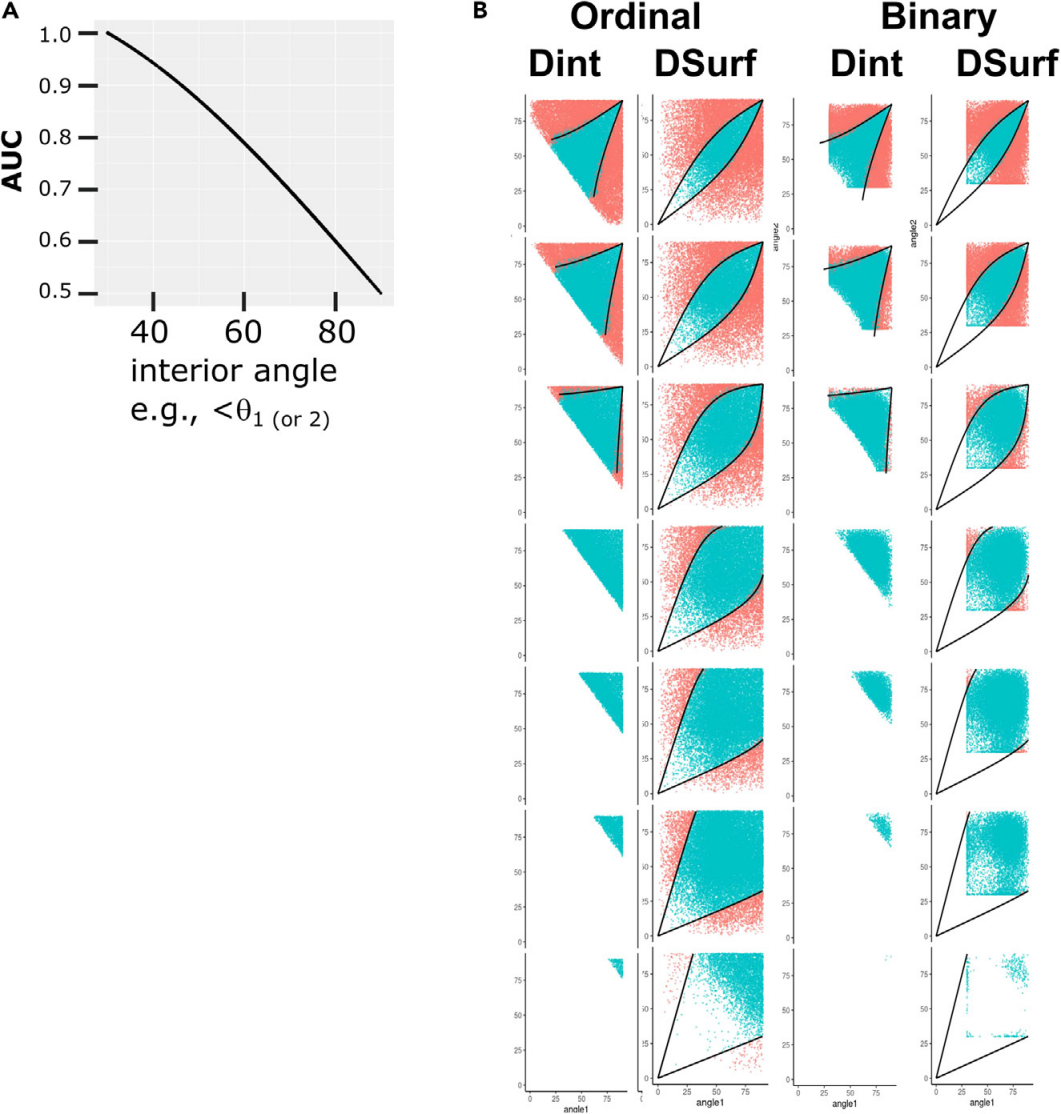
Comparison of DIRAC’s constraints on rank approximation vs. binary classification Here we show the bottom half of the simulated data from Figures 4 and 7. This presentation highlights those regions that are observable in the rank approximation context that are geometrically constrained/absent in the binary classification case.

If this artificial target vector is used in place of the barycenter-translated target vector used in the angle-based formulation of the DIRAC framework described in part III, then pairwise fusions selected to minimize the interior angle between this target vector and the fused system vector will automatically maximize the binary class separation, equivalent to maximizing the binary classification metric AUROC. To demonstrate the equivalence between binary classification AUROC and the interior angle to this artificial vector, we added the calculation of this angle to the DIRAC simulation framework used in our previous study and simulated a large number of ranking systems of all accuracy levels (from AUROC 0.5 to 1.0). We repeated the binary classification simulations performed in our previous work, and we show that using D_int_ and D_surf_ as measures of ***diversity***, and angle vs. binary artificial vector as ***accuracy***, a similar geometry of fusion is observed (similar relationship among input ***accuracy***, ***diversity***, and fusion ***accuracy***) (simulated data [Figures 6A–6C, D_int_, and Figures 6G–6I, D_surf_, and real-world [Figures 6D–6F, D_int_, and Figures 6J–6L, D_surf_). A focused comparison between the rank approximation and binary classification cases is plotted in Figure 7.

Although the plots in Figure 7 show that the geometric DIRAC framework performs equally well in this situation, the juxtaposition here of the binary classification and rank approximation results highlights an important difference, which is that there is an upper bound on the input system accuracies, resulting in empty rectangular regions in the binary classification plots. This results from the fact that, because of the restrictions on full rankings (i.e., they contain each integer from 1 to N exactly once), when they are treated as Euclidean vectors, they are subject to the geometric restriction that they are unable to point in exactly the same direction as a ranking system containing ties, including the artificial target vector constructed to represent the binary classification task. Therefore a “perfect” binary classifier vector will not be perfectly aligned with the artificial target vector, which is a necessary trade-off as there are potentially many such vectors (refer to Figure 1). If these were all perfectly aligned with the target vector, they would be perfectly aligned with each other (i.e., be the same), which is a contradiction.

## Discussion

Our previous study advanced quantitative understanding of the specific roles of ***accuracy*** and ***diversity*** in a broad, general class of system fusion problems but was subject to at least two critical limitations. Firstly, even though we were able to show that the DIRAC relationships/criteria applied independently of scoring system distribution, the target domain was restricted to binary classification, which is only a single example of the many possible target domains where one may wish to optimize performance using information fusion. Secondly, we were only able to describe the relationship in empirical terms, using large amounts of simulated data to build non-parametric models that captured the DIRAC relationships. This further tied the results to our specific choices of ***accuracy*** and ***diversity*** metrics (AUROC, PC, and SR) without revealing the structural/mathematical linkages between them. Although the solid empirical evidence that we provided indicated the existence of a potentially unknown phenomenon and suggested immediately useful and practical implementations, it could not adequately serve as a foundation to anchor deeper theoretical exploration of the principles underlying the DIRAC framework.

In the current study, we construct such a foundation by building on the conceptual skeleton (the ***accuracy***-***diversity*** relationship, and the importance of ranks) established in our initial study to remedy these shortcomings. To do this, we first construct an alternative formulation of pairwise system fusion in which the target domain is rank approximation rather than binary classification, with a system’s accuracy quantified by the difference from a single, optimal target ranking using Spearman rank correlation, rather than by the classification AUROC. We then established that the DIRAC criteria (***accuracy*** and ***diversity*** relationships) continued to hold in this new formulation via empirical examination similar to that in our previous study. Finally, we demonstrate that this formulation of pairwise system fusion may be described using well-known principles and constructs from combinatorics and symmetric group theory, allowing the systems involved, and the ***accuracy*** and ***diversity*** relationships linking them, to be described geometrically, with high precision, and at a generally applicable level. This result provides a precise mathematical representation of the concepts of ***accuracy*** (“good enough”) and ***diversity*** (“different enough”) in information fusion in which the synergistic roles relating these quantities are made explicit and quantitative, and explicitly provides a firm theoretical foundation for the DIRAC framework. We demonstrate the generality of this geometric formulation by using it to theoretically derive the empirical results we have observed, in both the rank approximation context earlier in this study, and in the binary classification context of our previous study.

Through this geometric lens, the empirical relationships that we observe linking input ***accuracy***, ***diversity***, and output ***accuracy*** are revealed to be a consequence of combinatoric structure at the level of the systems’ sample rankings, with both quantities represented by distances within the space in which these structures exist. This represents both ***accuracy*** and ***diversity*** as generally as possible, as they are uncoupled from any numerical influences because of the specific data generating processes and score distributions involved.

The use of the geometric framework underlying DIRAC also confers several other immediate, practical advantages compared with the empirical, simulation-based framework used previously. First and foremost is the more compact representation of the boundary (i.e., ΔSR_RF[AB]_ = 0 and its angle equivalent) between those fusions predicted to improve, and those fusions predicted not to improve. In our previous study, it was necessary to model this boundary non-parametrically for each required diversity value, which required the generation or continual storage of massive amounts of data to fully sample the possible values of input accuracy and diversity at random. Using this approach required a small range of diversity values to be specified, evenly spanning the value at which the boundary was to be calculated, to ensure an adequate density of points for the model; this overlap/noise at the boundary, clearly visible in our simulations, could also affect the model accuracy. As demonstrated in part III of the results section, the geometric formulation permits the exact, analytical calculation of the boundary, free from data storage, sampling sparsity, and non-parametric modeling issues.

From an applications standpoint, the expansion to rank approximation demonstrated that the DIRAC criteria relating input ***accuracy*** and ***diversity*** to output ***accuracy*** also apply when the target is a single ranking. In terms of immediate utility, this widens the range of data fusion problems/situations in which the DIRAC framework and criteria may be of use, from (balanced) binary classification only, to any problem where the target may be expressed as a desired ranking (or partial ranking). These include all balanced and unbalanced binary classification problems as well as multiclass classification problems. Rank approximation is a potentially useful goal in itself, if the objective is to rank patients by disease risk, for instance, or rank stocks by predicted performance/yield.

A direct benefit of the ability to use single rankings as a target is that it enables any information expressible as a vector to be used within the DIRAC framework; this broadens DIRAC’s applicability across different problem domains. We demonstrated above how to represent a binary variable by constructing an “artificial” ranking vector that expresses the perfect separation of two classes as a single directional axis through the permutahedron, and this intuition scales to higher dimensions.^42^^,^^43^ A ternary categorical variable forms a “triangular” plane (i.e., a simplex), defined by three maximally divergent vectors, each of which “points toward” perfect separation of one of the three possible categorical choices from the remaining two. The projection of any ranking system vector onto this plane will reveal the extent to which the system induces separation between those categories, and a normal vector to this plane (orthogonal to each component) represents rankings systems that are not associated with any such separation. Expressing such multiclass ordinal and non-ordinal targets using angles in the DIRAC framework is more involved than simple binary classification but requires no additional theory or mathematical machinery. Indeed, the exact same geometric machinery described earlier may be used to describe the results of any pairwise ranking system fusion, and, in this context, it is worth considering that all ranking systems (both input systems and target systems) have equivalent representations in this geometric framework.

This work increases the utility of the DIRAC framework when predicting fusions in unlinked datasets by removing the requirement for class labels when calculating ***diversity***. In our previous study, the DIRAC relationships were demonstrated using “within-class” correlation to measure ***diversity***. This correlation is analogous to the D_surf_ ***diversity*** measure used here; both measures remove any influence of the two input systems’ relative accuracies from the diversity between these two input systems, in the former case by averaging the correlation calculated within each of the two classes, and in the latter case by geometric elimination as discussed in part III of the results section. Unfortunately, both measures require knowledge of the class labels. In our previous study, we suggested that the ***accuracy*** and ***diversity*** that constitute the DIRAC criteria may be collected separately in space and time. If two models with known population-level accuracies were candidates for fusion in a new study, an independent “pilot” type study in which the class labels were known would be required to estimate the diversity, with the precision of this estimate (and therefore the overall prediction of fusion benefit) depending on the pilot study size. In the current study, we have shown that the needed ***diversity*** may be calculated without knowing the class labels. Empirical evidence of this was provided in parts I and III of the results section, in which SR and D_int_ were used effectively as measures of ***diversity*** in pairwise fusion, and theoretical justification was provided in parts III and IV, in which the equivalence between D_surf_ and D_int_ was demonstrated in the context of the geometric DIRAC formulation using spherical trigonometric identities. This equivalence effectively means that the ***diversity*** measure in the DIRAC criteria may be obtained in a population in which the class labels are *not* known. For any given dataset the correlation between two models may be calculated directly and used in the DIRAC criteria to assess the potential benefit of fusion, without “unblinding” any subset of the dataset, and without requiring the additional “pilot” type study that would be necessary otherwise. Removing the requirement for class labels when calculating ***diversity*** is potentially of great benefit when attempting to move previously established predictive or diagnostic models into a new population.

### DIRAC: Modeling at a different level

We propose that the DIRAC framework and the ***accuracy***/***diversity*** criteria it embodies offer a complementary way to work with and build upon existing information and conventional approaches in statistical modeling and machine learning. DIRAC’s complementary nature is best revealed by considering how the DIRAC framework is related to existing analytical approaches and representations of data.

A dataset is typically represented using a matrix or table-like structure, with columns representing features or measurements, and rows representing samples. The simplest spatial representation of such tabular data constructs a multidimensional space from the features in the [table/matrix] columns, with one independent, orthogonal axis per feature, and maps each sample to a point location in this space with the point coordinates for any given sample being its collection of observed values across all the features. In other words, the features define the space, and the samples correspond to locations within it; for our purposes here, we refer to this as the “primal” space. In the Multiethnic Cohort (MEC) dataset used above as a real-world example, the features are the DXA and MRI measurements (e.g., right upper arm fat mass) and samples are the individual subjects. In this example, the space is defined by these orthogonal imaging measurements, and the individuals are represented by points in this “primal” space.

The idea underlying predictive or descriptive modeling in this “primal” space is to find a more compact or task-specific representation of the structure of these points in this space. We may, for example, wish to model the linear dependence of the measurements within the sample population by fitting a linear regression through the points, or to discriminate between two different groups of sample points by finding a manifold that optimally separates them (e.g., using linear discriminant analysis [LDA] or singular value decomposition [SVD]). The majority of contemporary machine learning research focuses on these “primal” spaces, which are often massively multidimensional, and is devoted to the creation of methods for representing and leveraging the complex spatial structures of sample points within them.

This primal space is, however, not the only possible representation of a dataset. The other spatial representation of tabular data is created by swapping the roles of the columns and rows in the table. The columns are still features, and the rows are still samples, but, in this representation, the space is instead constructed using the samples (the rows), with one independent, orthogonal axis per sample, and the features map to point locations in this space, with the point coordinates for any given feature being its collection of values across all the samples. In other words, the samples define the space, and the features correspond to locations within it; we refer to this space as the “complementary dual” space to contrast it with the “primal” space defined above. In the MEC dataset used above as a real-world example, the features are, as noted above, the DXA and MRI measurements (e.g., right upper arm fat mass) and samples are the individual subjects. In this example, however, the space is defined by the individual subjects in the dataset, and the imaging measurements are represented by points in this complementary dual space. The complementary dual space is where the DIRAC framework and criteria operate.

It is immediately apparent, however, that modeling data within the complementary dual space is inherently complicated by a fundamental problem that prevents any meaningful interpretation of distance or spatial structure within this space. The problem is that the scales and units with which we measure are a property of the features, and not of the samples. This means that locating each feature point properly will require the whole set of sample axes to have different units for each point: for the height point, the sample axes need to represent centimeters, but for weight, they need to be kilograms, and for age, they need to be years. This means that, despite appearances, the complementary dual space spanned by the sample axes is not a common space in which these points can be located. We can still position these feature points according to their numerical coordinates, ignoring any units and scaling, but the distance between them, and therefore the spatial structure uniting them, will be meaningless. Given this problem, any attempt to extract information on the basis of distance or spatial structure is doomed to failure.

The work presented in this report demonstrates that the use of ranks resolves this problem. When the columns of the data matrix represent ranking systems, rather than scoring systems, then the distance along the sample axes in the complementary dual space represents the rank of that individual within the whole dataset, rather than the individual’s weight, or height, or any other specific unit. The space spanned by these axes, measuring rankings, is now one in which any set of features or measurements may be meaningfully compared, independently of the units and distributions of their numerical scores.

We have been implicitly using this “complementary dual” space throughout this paper, as it is the space throughout which the ranking systems we have discussed are distributed, and is therefore the space in which the DIRAC framework and criteria operate. Ranking systems without ties (which uniquely assign each of the full range of natural numbers from 1 to N to the samples) form a bounded subset of points within this space that corresponds to the vertices of the permutation polytope (i.e., S_N_ permutahedron) from the corresponding S_N_ symmetric group; this permutahedron is embedded in the complementary dual space as a convex manifold. There are other discrete points in this space that represent rankings that contain ties. These range from rankings containing a single tie (two individuals with the same rank), to rankings containing multiple tied values. One such multiply tied ranking is the “artificial” ranking constructed for our representation of binary classification; another, in the context of the “real-world” MEC example, would be liver fat percentage, if rounded to a pre-determined resolution to reflect instrumentation accuracy. These tie-containing ranking systems are not located on the permutahedron manifold because they do not represent proper permutations (in the combinatorics sense); rather, these points lie equidistant from the set of vertices that represent all possible ways to break the ties. Notably, integration of these points into the theory of combinatorics and symmetric group theory is limited. In the “complementary dual” space described here, however, all ranking systems, whether or not they contain ties, have equivalent spatial representation and are equally addressed via the use of angles, such as the ***accuracy*** angles (i.e., θ_1_ and θ_2_ in Figure 3F) and the ***diversity*** angles D_int_ and D_surf_ introduced in this report.

The results and ideas presented in this report have extended our previous system fusion results to encompass both rank approximation and binary classification, and have, more importantly, enabled the construction of a solid theoretical foundation able both to explain all empirical observations that we have made throughout our research in this area and to serve as a basis upon which to build as we continue to explore this area. We have shown/discussed how the space in which the approaches that we use arises via the concept of a “complementary dual” representation and that it is possible to do useful work in this space. Specifically, this representation allows the meaningful representation of the ***accuracy*** and ***diversity*** of model (ranking system) pairs as separate but related angle-based distances in a way that affords an accurate prediction of the outcome of their fusion. We have shown by demonstration that these angle-based geometric techniques are sufficiently general to work both for ranking system points without ties (located on the permutahedron manifold, demonstrated using rank approximation as an objective), and for ranking systems containing ties (not located on the permutahedron manifold, demonstrated using binary classification, in our last example). This geometric framework lends itself to formal, rigorous investigation of means to enhance fusions, e.g., the fusion of multiple model systems and the optimal weighting of input systems. We are actively pursuing these and additional expansions of the DIRAC framework (Sniatynski et al., manuscript in preparation and work in progress). Likewise, this provides one theoretical anchor to ground work examining how to integrate the information in scores into the DIRAC framework, potentially linking our other work in rank-score diversity^12^^,^^44^ and cognitive diversity^40^^,^^45^ with our work in DIRAC (here and Sniatynski et al.^19^). We believe that tools developed to make use of these ideas may prove to be complementary to existing statistical modeling and machine learning methods and may also be useful on their own.

## Experimental procedures

### Resource availability

#### Lead contact

Further information and requests for resources and reagents should be directed to and will be fulfilled by the lead contact, Bruce Kristal (bruce.kristal@tufts.edu).

#### Materials availability

This study did not generate new unique reagents.

#### Data and code availability

The accession number for the imaging data (reduced form only, no identifiers) reported in this paper is Zenodo: https://doi.org/10.5281/zenodo.5711898. The accession number for the simulation and figure generation code used in this paper (which includes the imaging data as a dependency) is available under a GPL version 3 open-source license at Zenodo: https://doi.org/10.5281/zenodo.10208051.^46^

### Simulations: Direct ranking system sampling using Gram-Schmidt orthonormalization

Given that we seek to perform pairwise fusions of ranking systems, where the target is another ranking system, we can avoid the Gaussian/exponentially modified Gaussian (EMG)-based sampling used in our previous paper and work directly with sampled ranking systems. To do this, the desired cardinality N (size of the dataset) of the systems is established (i.e., how many individuals/samples are in the population: how many samples are there to be ranked), and three random permutations of the vector [1,2,…,N] (i.e., the identity vector) are sampled. The Gram-Schmidt orthonormalization procedure is then employed to derive three mutually orthogonal basis vectors from these three random vectors, each of which is also orthogonal to the identity vector. We therefore have a 4-dimensional space, spanned by four orthogonal basis vectors (i.e., the three computed vectors, plus the identity vector).

We then sample random points from this space, and compute rankings from the points by weighting the ranking corresponding to each orthogonal axis by the location of the sampled point along that axis, summing over the four orthogonal axes for each ranked position, then re-ranking the result from 1 to N, with any ties broken arbitrarily. As the N increases, the chance that any two randomly sampled vectors will be correlated to any appreciable degree becomes vanishingly small, but by constraining the space of possible rankings as described above this problem is avoided. By treating the identity vector [1,2,…,N] as the target, and including this vector in the sampling space described above, ranking system points can be generated at a range of accuracies (depending on their sampled location along the target axis), and correlated with each other to an arbitrary extent (depending on their sampled locations along the other three axes). This accomplishes the same task as the Gaussian/EMG sampling and correlation induction scheme in our previous work, but it allows us to work with ranking systems directly.

The end result is a set of rankings that are correlated with the target to an extent ranging from perfectly to not at all (the full range of possible accuracies, given that the systems are oriented such that SRC≥0 with the target), and with each other similarly (from perfectly correlated to perfectly anti-correlated). This full range of accuracies and pairwise ($RS_{A}$ to $RS_{B}$) correlations is necessary to properly examine the performance of the DIRAC framework in the rank approximation context.

This ranking system construction methodology was used throughout this paper, either calculating the accuracy and diversity of a given system using Spearman rank correlation, or using angles, as described in the results section. For more comprehensive coverage of the sampling space, the random sampling and orthonormalization procedure was repeated multiple times throughout the construction of the simulated dataset, to prevent *all* the produced ranking systems from being sampled from exactly the same subspace. In practice, the sampling/orthonormalization was performed every 6th sample.

The correlation induction procedure used to alter the pairwise system correlations in our previous paper is not applicable to situations where the target is a single ranking (as in this rank approximation context), as opposed to a set of possible rankings. This is because, with a single target ranking, it is impossible to change the correlation between two systems without also changing their correlation with the target (i.e., their accuracy), leading to poor (and unpredictable) coverage of the possible sampling space.

### D_int_ boundary derivation algorithm

The objective in this case is to derive the $\left\langle \theta_{1},\theta_{2}\right\rangle$ pairs which fall exactly on the boundary separating fusions (i.e., see Figure 5 scatterplots, in which the relevant $\left\langle \theta_{1},\theta_{2}\right\rangle$ pairs fall directly on the black boundary lines that improve in accuracy from those that do not improve, when using $D_{\mathrm{int}}$ to quantify pairwise diversity. Because we are using pairwise mean fusion, such a point necessarily corresponds with the geometric situation shown in Figure 5, with $\theta_{1}$ (the accuracy of the better system, i.e., the one closer to the target point $RS_{T}$) exactly equal to $\theta_{M}$, which is the “accuracy” angle separating the target point $RS_{T}$ and the midpoint, M, which is the midpoint of the geodesic connecting $RS_{A}$ and $RS_{B}$. This geometric system can be simplified into two right spherical triangles by the definition of another point, Q, located at the geodesic midpoint between M and $RS_{A}$ (necessarily, because $\theta_{1}$ must equal $\theta_{M}$ at the boundary). The “accuracy” angle between the point Q and the target $RS_{T}$ is denoted $\theta_{Q}$.

The geometry of this situation suggests a simple, iterative approach for generating a set of $\left\langle \theta_{1},\theta_{2}\right\rangle$ points at any desired resolution, for any value of the pairwise diversity $D_{\mathrm{int}}$. This is done by fixing $D_{\mathrm{int}}$ to the desired value and varying the angle $\theta_{Q}$. The spherical cosine identities then give unique boundary coordinates $\left\langle \theta_{1},\theta_{2}\right\rangle$ for any value of $\theta_{Q}$.

**Figure 8:**
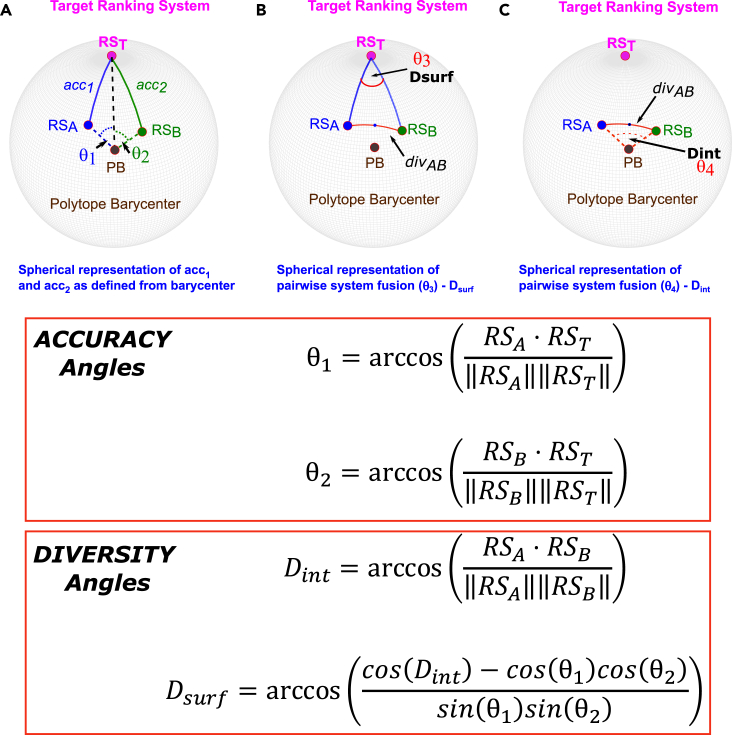
Reproduction of Figure 3 to be used as reference for methods For (A), (B), and (C), please see the legend for Figures 3F, 3G, and 3H, respectively.

All accuracy and diversity angles are calculated from the input ranking system vectors ($RS_{A}$ and $RS_{B}$) and the target vector ($RS_{T}$) using the spherical trigonometric identities above (Figure 8).

Algorithm parameters:

(1) Interior angle diversity, $D_{\mathrm{int}}\in \mathbb{R}$
(2) Resolution, N∈N

Initialize:

(1) Calculate step size, ss=πN
(2) Initialize $\theta_{Q}$ vector, $\theta_{Q}=\left[0,i\cdot ss,\ldots \;|\;i\;\to \;1\;\ldots \;N\right]$

For each value v in vector $\theta_{Q}$:

(1) $\theta_{1}\;\leftarrow \arccos\left(\;\cos\left(v\right)\cdot \cos\left(\frac{1}{4}\cdot D_{\mathrm{int}}\right)\right)$
(2) $\theta_{2}\;\leftarrow \arccos\left(\;\cos\left(v\right)\cdot \cos\left(\frac{3}{4}\cdot D_{\mathrm{int}}\right)\right)$
(3) Return boundary coordinate, $\left\langle \theta_{1},\theta_{2}\right\rangle$

Result: vector of boundary coordinates, $\left[\;{\left\langle \theta_{1},\theta_{2}\;\right\rangle }_{1},{\left\langle \theta_{1},\theta_{2}\;\right\rangle }_{2},\ldots ,{\left\langle \theta_{1},\theta_{2}\;\right\rangle }_{N}\;\right]$

### D_surf_ boundary derivation algorithm

The boundary derivation is more complicated in the situation where $D_{surf}$ is being used to quantify pairwise diversity, rather than $D_{\mathrm{int}}$. On the boundary we still need $\theta_{1}$ and $\theta_{M}$ to be exactly equal to each other, but because the angle $D_{\mathrm{int}}$ is not known, and is not derivable given only $D_{surf}$ and $\theta_{1}$, the right spherical triangle decomposition used above will not help, and we cannot easily derive a closed-form solution for the corresponding boundary coordinate value of $\theta_{2}$.

However, given any two input system accuracies ($\theta_{1}$ and $\theta_{2}$) and a fixed $D_{surf}$ diversity value, we can use the spherical trigonometric identities above to check whether the boundary conditions hold, and if they do not, determine on which side of the boundary the point $\left\langle \theta_{1},\theta_{2}\right\rangle$ will fall (as above, see Figure 5). To do this, the values of $\theta_{1}$, $\theta_{2}$, and $D_{surf}$ are first used to calculate the value of $D_{\mathrm{int}}$ that would need to hold in this situation. The $D_{\mathrm{int}}$ value corresponds to the length of the geodesic connecting $RS_{A}$ to $RS_{B}$, and if we assume that boundary conditions hold, we can divide its length into quarters as above (i.e., finding the geodesic midpoint M, and the right-angle point Q), and again use the right spherical triangle decomposition to calculate the midpoint accuracy $\theta_{M}$, which again must be equal to $\theta_{1}$ as is required for boundary conditions to apply. If $\theta_{1}=\theta_{M}$, then the point $\left\langle \theta_{1},\theta_{2}\right\rangle$ falls exactly on the boundary. If $\theta_{1}>\theta_{M}$ then $\theta_{2}$ is not large enough relative to $\theta_{1}$, and the point falls inside the boundary. Conversely if $\theta_{1}<\theta_{M}$ then $\theta_{2}$ is too large relative to $\theta_{1}$, and the point falls outside the boundary.

With these mathematical relationships defined, we can use an iterative procedure to find a point $\left\langle \theta_{1},\theta_{2}\right\rangle$ that is arbitrarily close to the boundary. We do this by selecting a desired value for the more accurate input ranking system, $\theta_{1}$, setting $\theta_{2}=\theta_{1}$ initially, and increasing $\theta_{2}$ in increments, using a specified step size t, and performing the boundary-crossing test described above after each increment. When the test indicates the boundary has been crossed, we backtrack to the previous $\theta_{2}$ value: the upper bound on the distance separating this point from the boundary will be the step size used (t). Setting the step size arbitrarily small will find a point arbitrarily close to the boundary, but it may be very slow for very small step sizes. To speed up the process, we can use a “binary search” style approach, where we first increment $\theta_{2}$ using very coarse steps until we first cross the boundary, then backtrack to the previous $\theta_{2}$ value and continue incrementing from that point with a smaller step size. Many rounds of this procedure may be performed, allowing an arbitrarily close approach to the boundary without incurring a significant performance penalty.

We must then iterate this iterative procedure for each value of $\theta_{1}$ for which we want to find the corresponding boundary coordinate $\theta_{2}$. The overall resolution of the calculated boundary curve is established by the selection of $\theta_{1}$ points, as in the $D_{\mathrm{int}}$ boundary derivation case.

All accuracy and diversity angles are once again calculated from the input ranking system vectors ($RS_{A}$ and $RS_{B}$), and the target ranking system $RS_{T}$, using the spherical trigonometric identities above.

Algorithm parameters:

(1) Surface angle diversity, $D_{surf}\in R$ (radians)
(2) Resolution, N∈N
(3) Iterative search tolerance, t∈R

Initialize:

(1) Calculate step size, ss=πN
(2) Initialize $\theta_{1}$ vector, $\theta_{1}=\left[0,i\cdot ss,\ldots \;|\;i\;\to \;1\;\ldots \;N\right]$

For each value v in vector $\theta_{1}$:

(1) $\theta_{1},\theta_{2}\leftarrow v$ (set both accuracies equal to v)
(2) Until boundary crossed:
  (a) $\theta_{2}\leftarrow \theta_{2}+t$
  (b) $\text{Derive}\;D_{\mathrm{int}}\text{:}\;D_{\mathrm{int}}=\arccos\left(\cos\left(D_{surf}\right)\sin\left(\theta_{1}\right)\sin\left(\theta_{2}\right)+\cos\left(\theta_{1}\right)\cos\left(\theta_{2}\right)\right)$
  (c) Calculate ${\;\theta }_{M}$ the $\theta_{1}$ accuracy necessary assuming boundary geometry, given values of $\theta_{2}$ set in step a. and $D_{\mathrm{int}}$ derived in step b: $\theta_{M}=\arccos\left(\frac{\cos\left(\theta_{2}\right)\cos\left(\frac{1}{4}\cdot D_{\mathrm{int}}\right)}{\cos\left(\frac{3}{4}\cdot D_{\mathrm{int}}\right)}\right)$
  (d) If $\theta_{M}\geq \theta_{1}$, goto 3, else resume loop at 2
(3) Return boundary coordinate, $\left\langle \theta_{1},\theta_{2}\right\rangle$

Result: vector of boundary coordinates, $\left[\;{\left\langle \theta_{1},\theta_{2}\;\right\rangle }_{1},{\left\langle \theta_{1},\theta_{2}\;\right\rangle }_{2},\ldots ,{\left\langle \theta_{1},\theta_{2}\;\right\rangle }_{N}\;\right]$

The “earth” analogy may help clarify the relationships. In this analogy, RS_T_ is the equivalent of the North Pole, with the two systems, RS_A_ and RS_B_, being the equivalent of two cities, e.g., London (51.5072° N, 0.1276° W, defined as RS_A_, as it is closer to the North Pole) and a second city (CITY2), further south, which we define as RS_B_). In this scenario, θ_1_ is the angle at the Earth’s center with London and the North Pole as the “ends” on the Earth’s surface. θ_2_ is the angle at the Earth’s center with CITY2 and the North Pole as the “ends” on the Earth’s surface. D_surf_, θ_3_, is the difference in longitude between London and CITY2. D_int_, θ_4_, is the angle at the Earth’s center with London and CITY2 as the “ends” on the Earth’s surface. θ_m_ is the angle at the Earth’s center with the great circle’s midpoint and the North Pole as the “ends” on the Earth’s surface. The situation where θ_m_ = θ_1_ defines the boundary condition for where or not a fusion will be beneficial. Thus, in this example, when point M (the pairwise fusion, assuming no ties) sits at the same latitude (i.e., “accuracy”) as the more northern city, a fusion would be neutral, and when θ_1_ > θ_m_ the fusion will be beneficial.

Note the point Q is used for simplifying calculations, and has no direct interpretative meaning in the fusion. For those interested, it may be considered as the point on the great circle route one-quarter of the distance between the cities. At the boundary between positive and negative fusions, the surface angle at Q (between target and RS_A_ or target and RS_B_) will be 90°. This makes it a right spherical triangle. This simplifies the math and enables direct use of the identities above.

### Fusion

The process of fusion creates a single, new ranking system from two input ranking systems. As per our previous report, we here examined the simplest type of fusion, which simply averages the value of the two input ranking systems in a pairwise fashion.

### LOWESS curves

As per our previous report, LOWESS curves were created in R version 3.5.2. Here they were trained on the rank fusion data shown in Figure 2.

### Real data: DXA/MRI data from MEC-APS

As per our previous report, we demonstrated the applicability of the fusion techniques presented in this work to real-world data using MRI-based measures of body fat distribution (e.g., liver fat, visceral fat at the L1-L2 vertebral boundary; see supplemental information for full list) as the ground-truth target variables, and DXA variables that captured general body fat serving as the predictor variables to fuse. In the original report, we used quantile data, here we used individual rankings (with ties broken stochastically). These data were drawn from the MEC-APS, in which 1,861 individuals from the MEC had their body composition measured by DXA and their abdominal fat distribution assessed by MRI between L1 and L5 from 2013 to 2016. This study was institutional review board (IRB) approved. Data from the initial 1,000 participants recruited were used. Details on the MEC itself and the imaging study have been published, but these are not critical for the current study.^29^

We used each available MRI variable as a target and determined the SR (Figure 2) or its angle equivalent (Figures 4, 6, and 7) between this target and each DXA predictor variable. Variables are oriented to have non-negative correlations with the target (by multiplying by −1 if needed). We evaluated fusions using all possible pairs of DXA variables and determined the accuracy of the fused system. The relationship between the accuracies of the input systems, their diversity, and the gain or loss in accuracy with fusion was then compared with that of the simulated data.

## Supplemental information

Document S1. Data S1 and S2 and Figures S1–S9

Data S3. Interactive simulator allowing rotation of representations of the S2, S3, and S4 permutahedra

Data S4. Interactive simulator allowing rotation of representations of the S2, S3, and S4 permutahedra Differs from Data S3 with respect to shading.

Data S5. Data simulation and fusion assessment framework

Document S2. Article plus supplemental information
