# Supplementary material for: The DIRAC framework: Geometric structure underlies roles of diversity and accuracy in combining classifiers
Source: Patterns (N Y). 2024 Feb 5;5(3):100924. doi: 10.1016/j.patter.2024.100924 (PMC10935508; doi:10.1016/j.patter.2024.100924)

Patterns, Volume *5*

Supplemental information

The DIRAC framework:

Geometric structure underlies roles of *DIVERSITY*

and *ACCURACY* in combining classifiers

Matthew J. Sniatynski, John A. Shepherd, Lynne R. Wilkens, D. Frank Hsu, and Bruce S. Kristal

Supplemental Information

Data S1: DXA Variables (31 Total):……..…… 2

Data S2: MRI variables (39 Total):…………… 4

Figures S1 - S9 …………………………………. 7

Data S1: DXA Variables (31 Total):

Note: variables having the corr tag were corrected for differences in minor analytical differences in instrumentation between sites/vendors. Using these variables, and their cognate, pre-correction values, shows that such systematic noise is neither fixed by DIRAC nor does it compromise DIRAC. DIRAC operates at the level of system fusion, which is beyond this and is thus blind/neutral to these types of noise, other than the expectation that corrected models will have higher accuracy, and DIRAC utilizes this higher accuracy.

Global metrics

DXA_mass_tot_corr	Total body mass, corrected
DXA_mass_tot	Total body mass

Fat Metrics

DXA_fat_tot_corr	Total fat mass in body, corrected
DXA_fat_tot	Total fat mass in body
DXA_fat_trunk	Fat mass in trunk area
DXA_fat_arms	Fat mass in arms
DXA_fat_legs	Fat mass in legs
DXA_fat_android	Fat mass in android portion of body
DXA_fat_gynoid	Fat mass in gynoid portion of body
DXA_tfat_area	Total abdominal cross-sectional fat area (sfat+vfat)
DXA_sfat_area	Abdominal subcutaneous fat cross sectional area
DXA_vfat_area	Visceral fat cross sectional area

Fat Ratios

DXA_fat_android_gynoid	Ratio of fat in the android to the gynoid portion of the body
DXA_fat_trunk_limb	Ratio of fat in trunk to fat in limbs
DXA_pfat_tot_corr	Percent body fat, corrected
DXA_pfat_tot	Percent body fat
DXA_pfat_trunk	Percent fat in trunk region

Lean Metrics

DXA_lean_tot_corr	Total lean body mass, corrected
DXA_lean_tot	Total lean body mass
DXA_lean_trunk	Total lean mass in trunk
DXA_muscle_tot	Total muscle mass
DXA_lean_gynoid	Lean mass in gynoid portion of body
DXA_lean_android	Lean mass in android portion of body
DXA_lean_arms	Lean mass in arms
DXA_lean_legs	Lean mass in legs

Bone Metrics

DXA_BMC_tot_corr	Total bone mineral content, corrected
DXA_BMC_tot	Total bone mineral content
DXA_BMD_tot_corr	Total bone mineral density, corrected
DXA_BMD_tot	Total bone mineral density
DXA_BMD_pelvis	Bone mineral density, pelvis
DXA_BMD_spine	Bone mineral density, spine

Data S2: MRI variables (39 Total):

Control variable

OMRI_weight	Weight of subject at time of MRI

Liver Fat

OMRI_pct_liver_fat	Percent of the liver that is fat

Slice-Based fat Determinations

The remaining MRI variables were either assessed in each of four slices or represent the average of those four values. Thus:

L1-L2 refers to the MRI slice better the L1 and L2 vertebrae

L2-L3 refers to the MRI slice better the L2 and L3 vertebrae

L3-L4 refers to the MRI slice better the L3 and L4 vertebrae

L4-L5 refers to the MRI slice better the L4 and L5 vertebrae

OMRI_abdo_area_L1L2	Abdominal area at a given slice
OMRI_abdo_area_L2L3	
OMRI_abdo_area_L3L4	
OMRI_abdo_area_L4L5	

OMRI_pct_abdofat_L1L2	Percent of the abdominal area at a given slice that is fat
OMRI_pct_abdofat_L2L3	
OMRI_pct_abdofat_L3L4	
OMRI_pct_abdofat_L4L5	

OMRI_abdofat_L1L2	Abdominal area at a given slice that is fat
OMRI_abdofat_L2L3	
OMRI_abdofat_L3L4	
OMRI_abdofat_L4L5	
OMRI_viscfat_L1L2	Abdominal area at a given slice that is visceral fat
OMRI_viscfat_L2L3	
OMRI_viscfat_L3L4	
OMRI_viscfat_L4L5	
OMRI_viscfat_avg	Average of the above four values
OMRI_subcfat_L1L2	Abdominal area at a given slice that is subcutaneous fat
OMRI_subcfat_L2L3	
OMRI_subcfat_L3L4	
OMRI_subcfat_L4L5	
OMRI_subcfat_avg	Average of the above four values
OMRI_pct_viscfat_L1L2	Percent of the fat in a given abdominal slice that is visceral
OMRI_pct_viscfat_L2L3	
OMRI_pct_viscfat_L3L4	
OMRI_pct_viscfat_L4L5	
OMRI_pct_viscfat_avg	Average of the above four values
OMRI_pct_subcfat_L1L2	Percent of the fat in an abdominal slice that is subcutaneous
OMRI_pct_subcfat_L2L3	
OMRI_pct_subcfat_L3L4	
OMRI_pct_subcfat_L4L5	
OMRI_pct_subcfat_avg	Average of the above four values

OMRI_viscfat_subcfat_L1L2	Ratio of visceral to subcutaneous fat in a given slice
OMRI_viscfat_subcfat_L2L3	
OMRI_viscfat_subcfat_L3L4	
OMRI_viscfat_subcfat_L4L5	
OMRI_viscfat_subcfat_avg	Average of the above four values

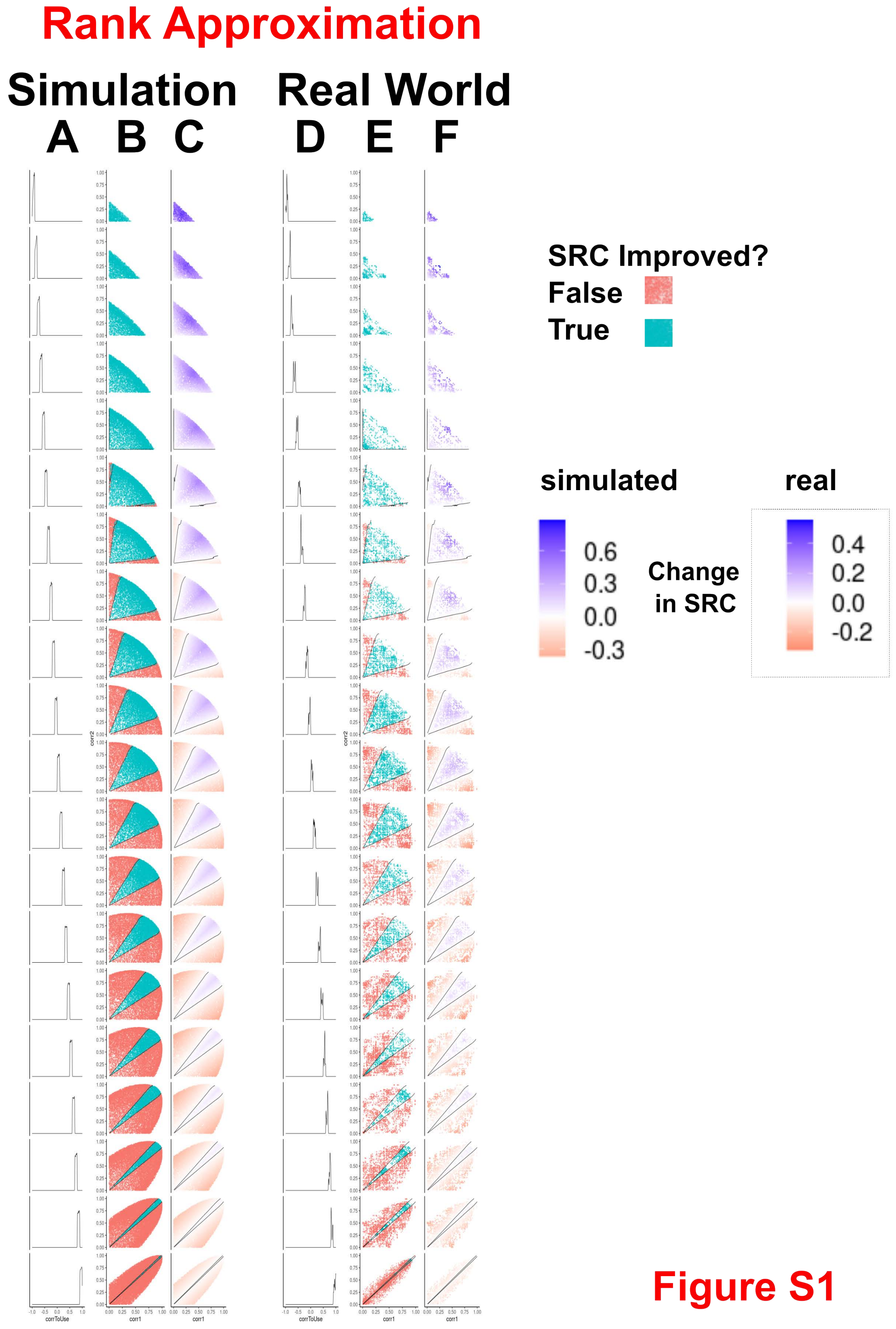

Rank Approximation
Simulation
Real World
A
B
C
D
E
F
SRC Improved?
False
True
simulated
real
Change in SRC
0.6
0.3
0.0
-0.3
0.4
0.2
0.0
-0.2
corrToUse
corr1
corr2

Figure S1

Rank Approximation

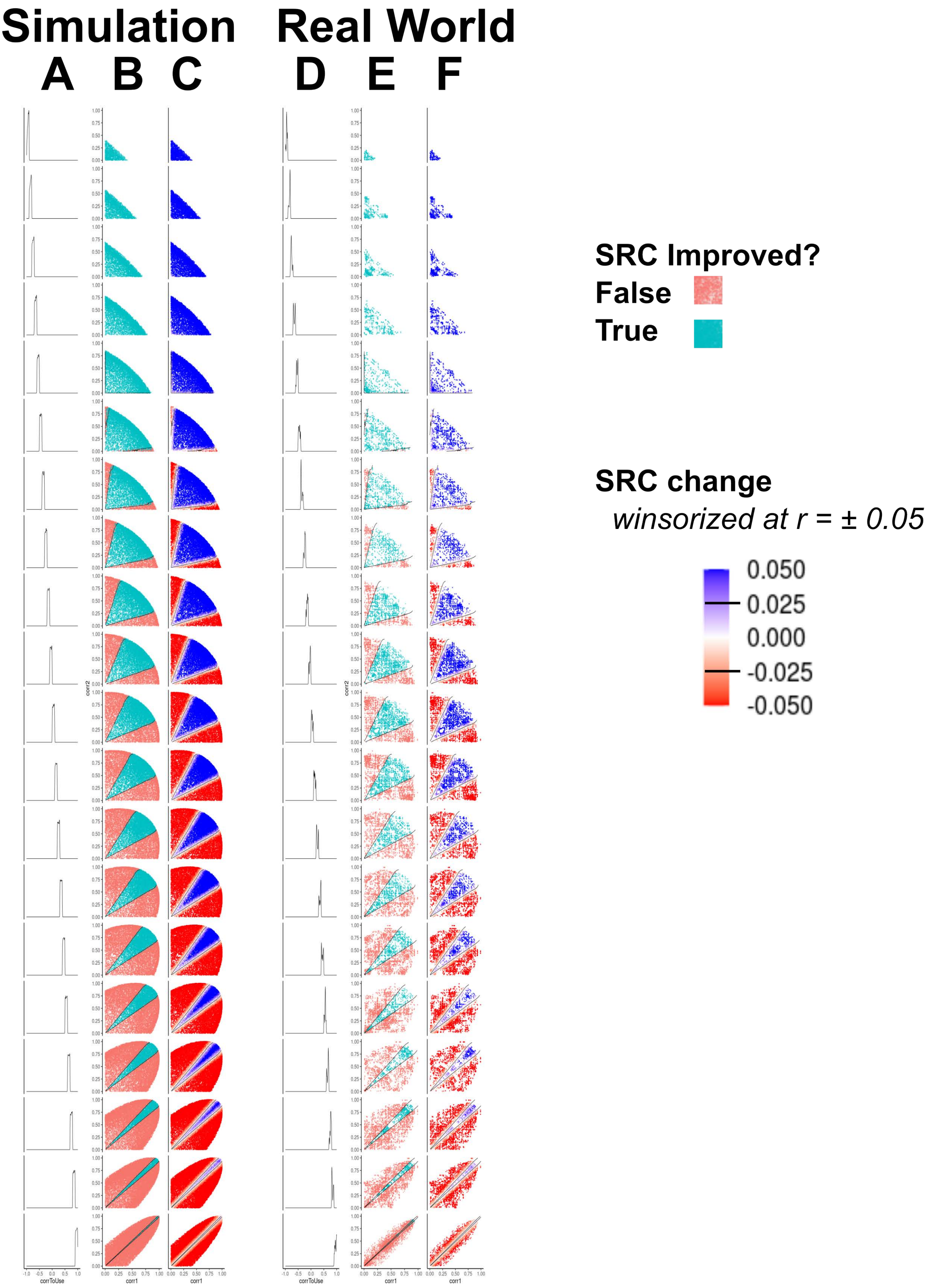

Figure S2

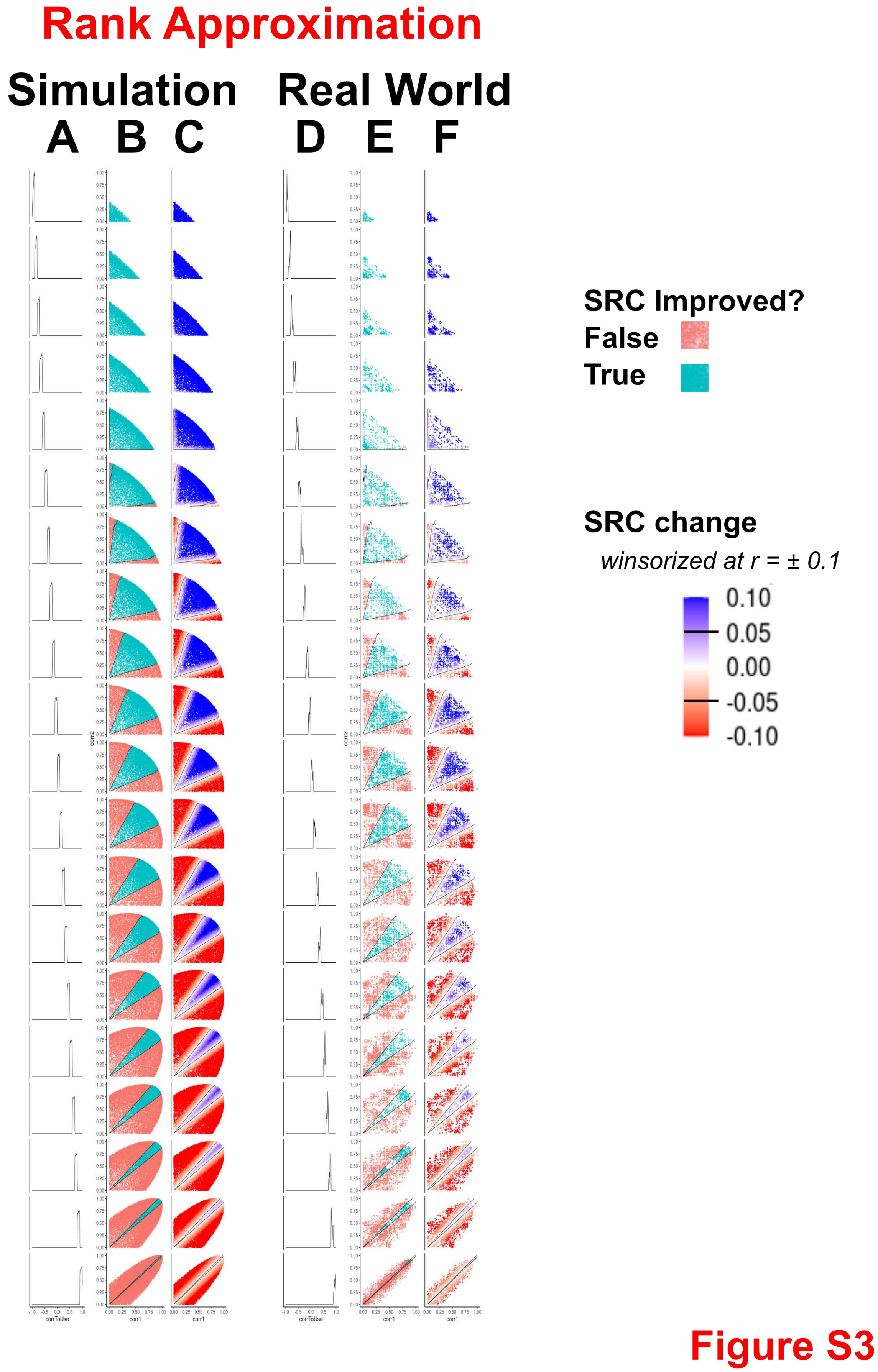
Rank Approximation
Simulation
Real World
A
B
C
D
E
F
SRC Improved?
False
True
SRC change
winsorized at r = ± 0.1
0.10
0.05
0.00
-0.05
-0.10
corrToUse
corr1
corr2
Figure S3

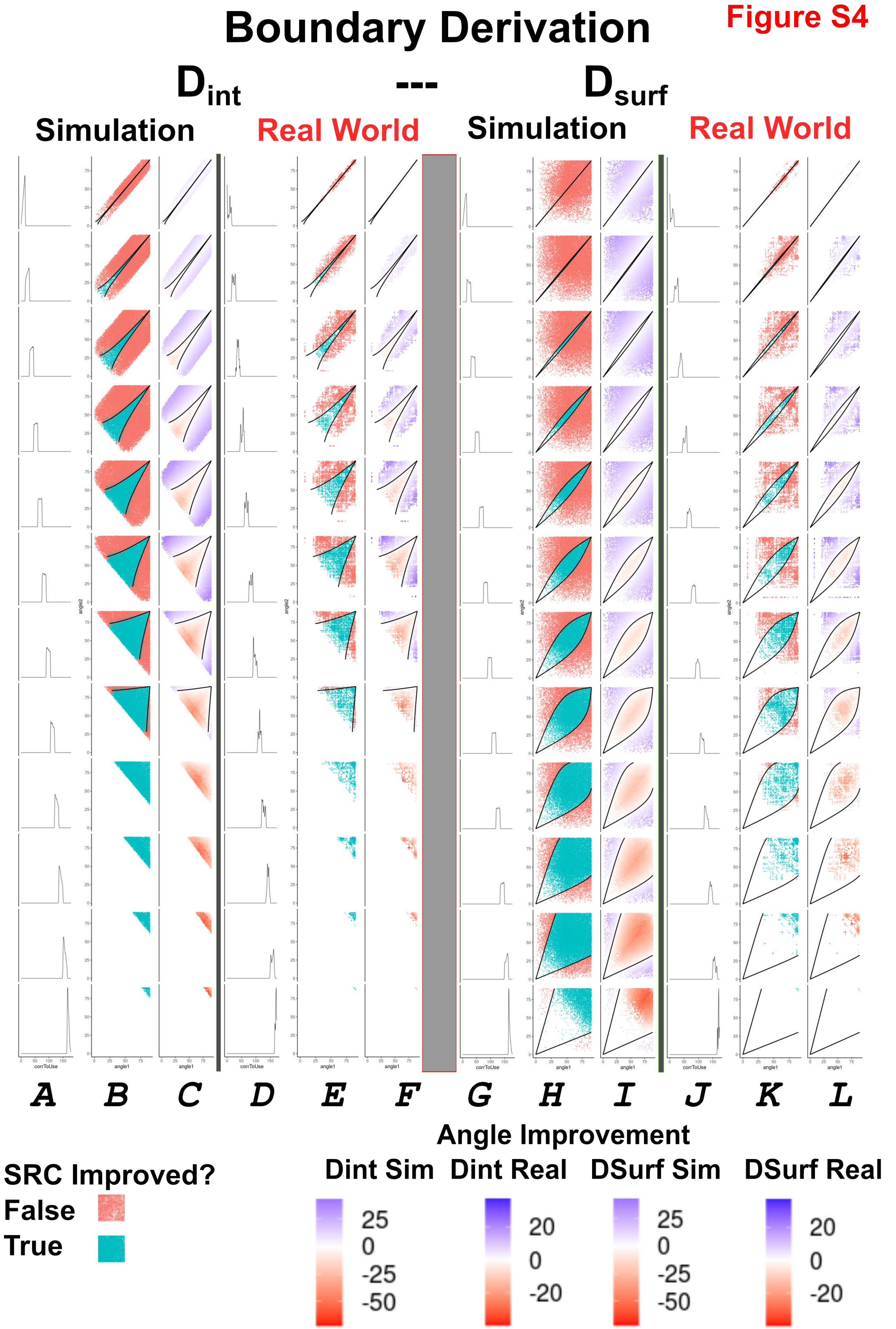
Figure S4
Boundary Derivation
D int --- D surf
Simulation
Real World
Simulation
Real World
angle1
angle2
corrToUse
A
B
C
D
E
F
G
H
I
J
K
L
Angle Improvement
Dint Sim
Dint Real
DSurf Sim
DSurf Real
SRC Improved?
False
True
25
0
-25
-50
20
0
-20

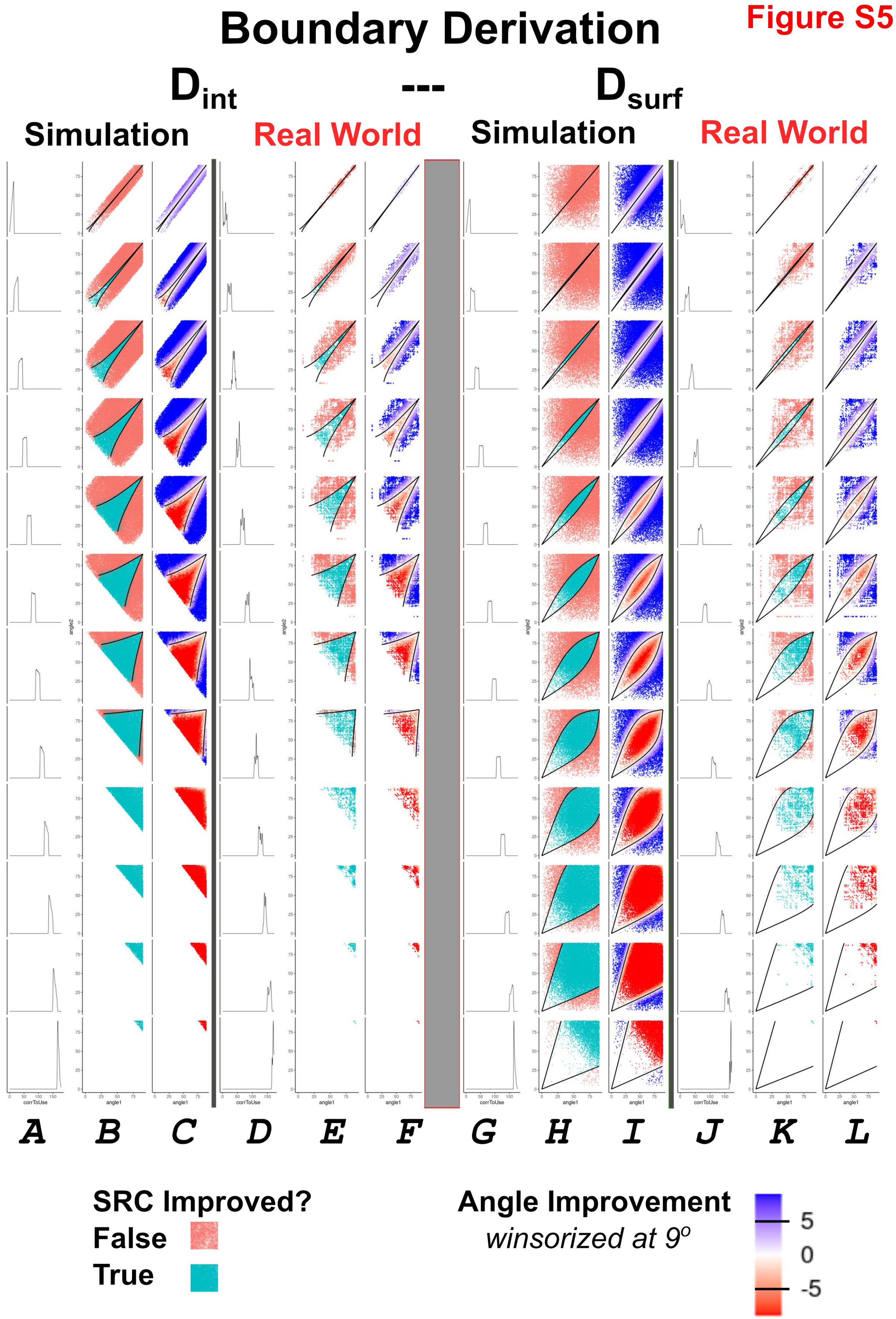
Figure S5
Boundary Derivation
D_{int} --- D_{surf}
Simulation
Real World
Simulation
Real World
corrToUse
angle1
angle2
A B C D E F G H I J K L
SRC Improved?
False
True
Angle Improvement
winsorized at 9°
5
0
-5

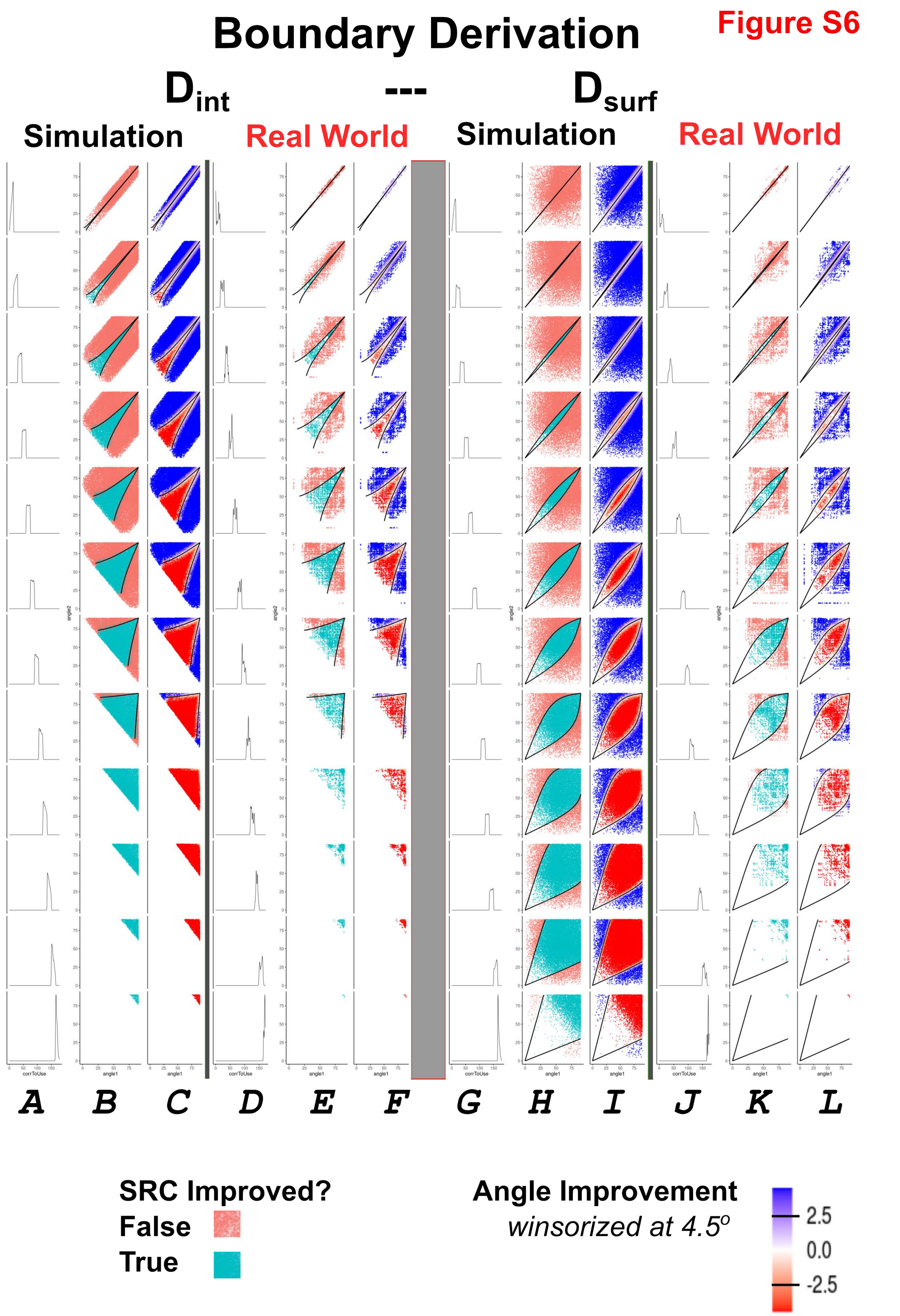
Figure S6
Boundary Derivation
D_{int} --- D_{surf}
Simulation
Real World
Simulation
Real World
corrToUse
angle1
angle2
A B C D E F G H I J K L
SRC Improved?
False
True
Angle Improvement
winsorized at 4.5°
2.5
0.0
-2.5

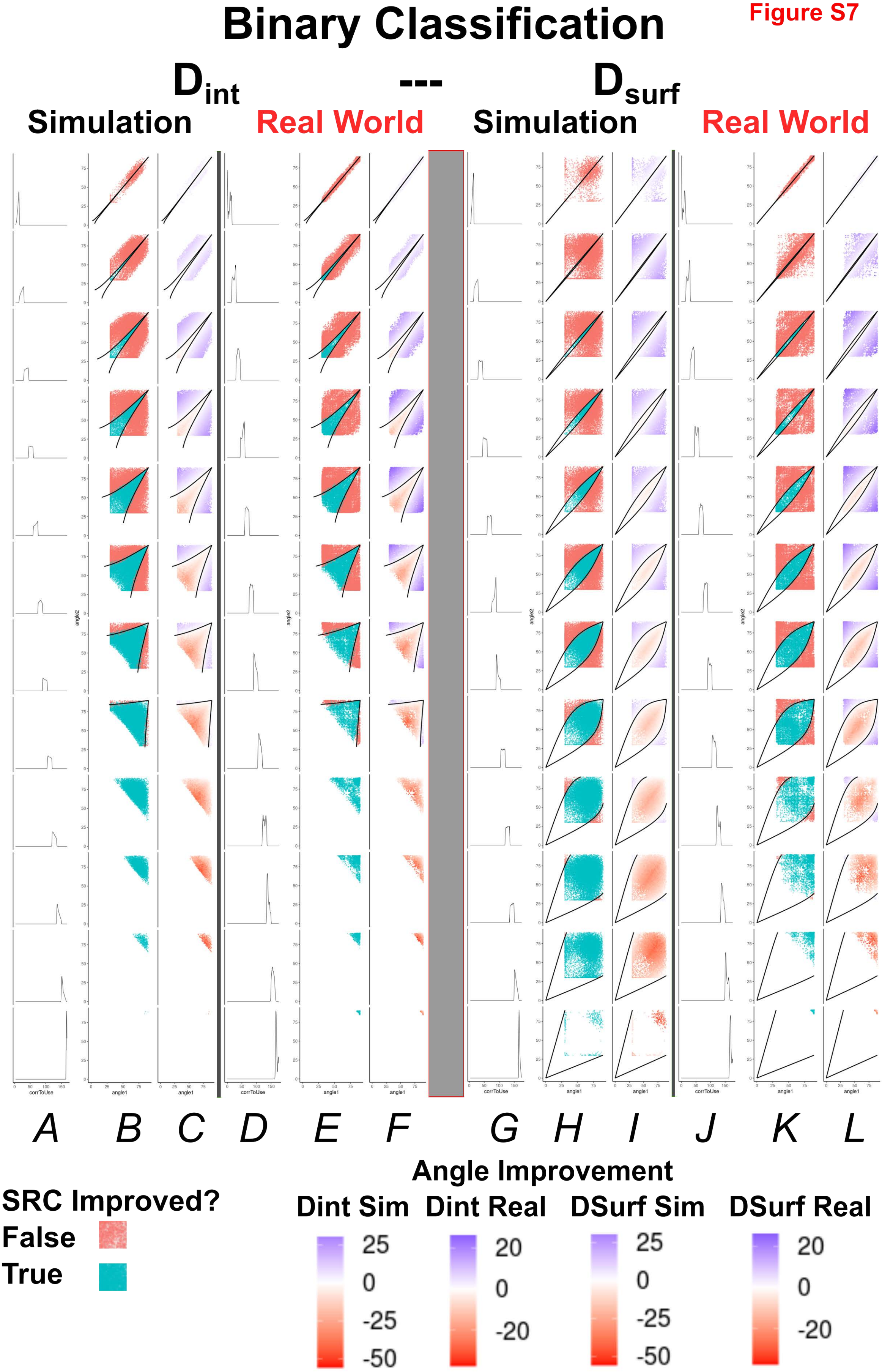

Figure S7
Binary Classification
Dint

Dsurf
Simulation
Real World
Simulation
Real World
angle2
angle1
corrToUse
A B C D E F G H I J K L
SRC Improved?
False
True
Angle Improvement
Dint Sim
Dint Real
DSurf Sim
DSurf Real
25
0
-25
-50
20
0
-20

Binary Classification

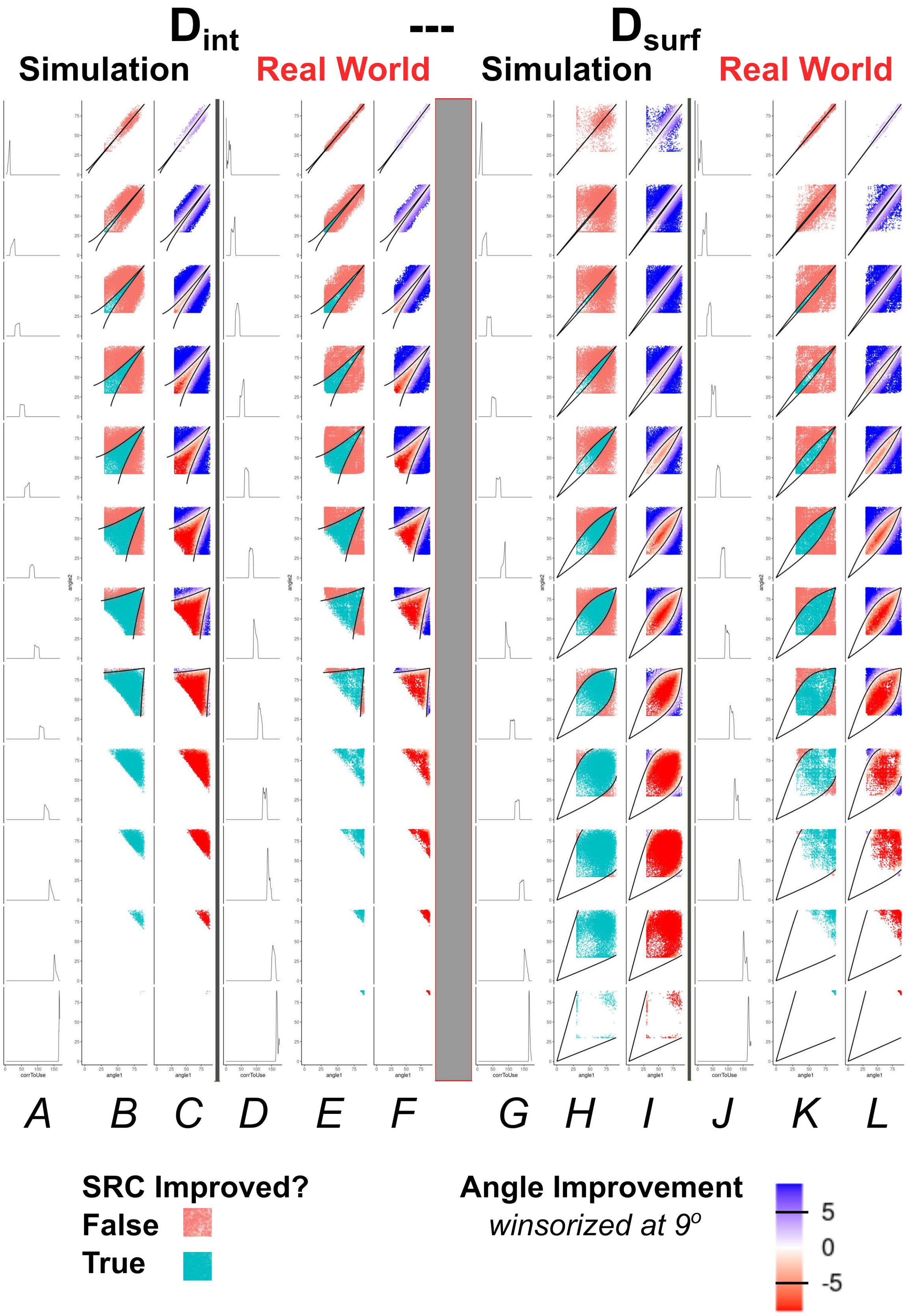

Binary Classification

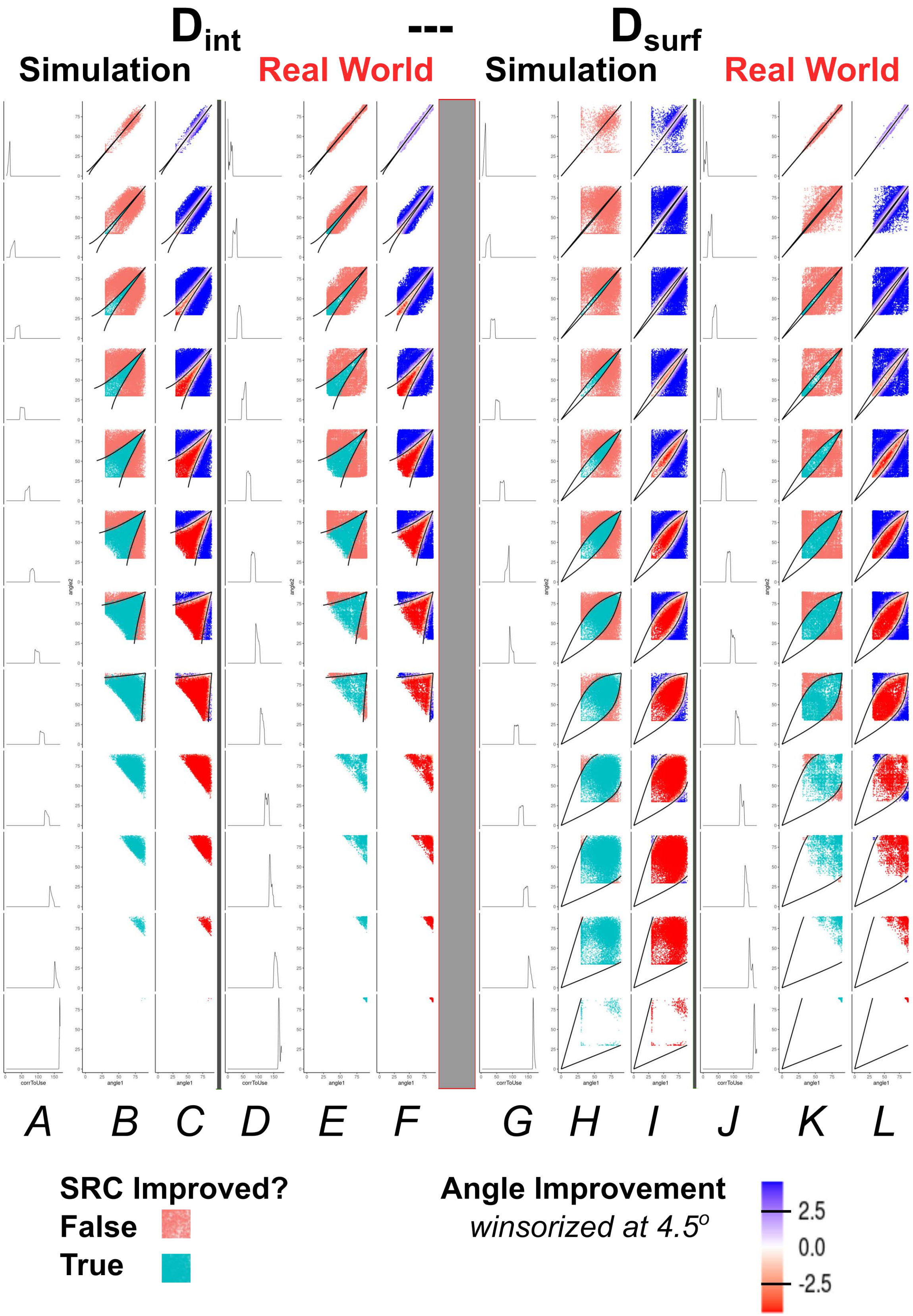

Supplement: Document S1. Data S1 and S2 and Figures S1–S9 [file mmc1.pdf]
