# Supplementary material for: The DIRAC framework: Geometric structure underlies roles of diversity and accuracy in combining classifiers
Source: Patterns (N Y). 2024 Feb 5;5(3):100924. doi: 10.1016/j.patter.2024.100924 (PMC10935508; doi:10.1016/j.patter.2024.100924)
Supplement: Data S3. Interactive simulator allowing rotation of representations of the S2, S3, and S4 permutahedra [file mmc2.zip › patter_00100924_PATTERNS-D-23-00126 - Simulator with labeled S4.html]

RGL Graphics for Representable Permutahedra


# RGL Graphics for Representable Permutahedra

These are interactive graphics, dragging on them with the mouse (in a relatively intuitive way) will rotate them around, and the scroll wheel will zoom in and out (I think).

## S2 Permutahedron (in 3D)

## S3 Permutahedron (in 3D)

## S4 Permutahedron (in 3D)

## S4 Permutahedron (in 3D), with labels

The labels here correspond to the 4D coordinates of each vertex. Because a permutation may be specified by using only N-1 rankings (because the last ranking is implicitly specified by knowing all the others), The full 4 dimensions are not needed to visualize the polytope, and the “extra” dimension may be removed by a PCA projection, as done here.

In the case of the S3 hexagon above this is more readily visible – the hexagon itself is only a 2 dimensional shape, but the coordinates of each point require all 3 dimensions to specify. If we simply plotted an even hexagonal shape on a 2D plane, then labeled the vertices with the 3D coordinates, that would be the equivalent of what we’ve done here.

End of test.
