# Supplementary material for: The DIRAC framework: Geometric structure underlies roles of diversity and accuracy in combining classifiers
Source: Patterns (N Y). 2024 Feb 5;5(3):100924. doi: 10.1016/j.patter.2024.100924 (PMC10935508; doi:10.1016/j.patter.2024.100924)
Supplement: Data S5. Data simulation and fusion assessment framework [file mmc4.zip › SupportingCode_DIRAC_02/Figure_05/surface_boundary_dataIncluded.html]

System 1 Accuracy:

Diversity:

ScatterPlot Slice Thickness:
